# Supplementary material for: Differences in Cannabis and Cannabidiol Attitudes, Perceptions, and Behaviors Between US Adolescents Receiving Mood Disorder Treatment and Their Parents Across Legal Contexts
Source: Int J Environ Res Public Health. 2025 Oct 16;22(10):1576. doi: 10.3390/ijerph22101576 (PMC12563936; doi:10.3390/ijerph22101576)
Supplement: Supplementary file 1 [file ijerph-22-01576-s001.zip › ijerph-3801705-supplementary.pdf]

Supplementary Online Content

Hammond CJ, Fristad MA, Moon YJ, Batt MM, Sullivan AE, Schneck CD, Ghaziuddin N, Dopp R, Leffler JM, Singh MK, Weinstein S, Hulvershorn L, Miller L. Differences in cannabis and cannabidiol related attitudes, perceptions, and behaviors between US adolescents receiving mood disorder treatment and their parents across states with and without cannabis legalization. *International Journal of Environmental Research and Public Health* 2025

Table of Contents:

Supplemental eMethods S1. MABS survey development and item details

Supplemental eMethods S2. Measures and Variables Used in Statistical Analysis

Supplemental eTable S1. MCL and RCL Effective Dates by State, and Cannabis Policy Details for each US state in the Sample

Supplemental eResults S1. Description of Neutral and Uncertain Response Outcomes from Main analyses

Supplemental eTable S2. Main Analysis Results: Multinominal and Linear Regression Models Examining Marijuana-related Attitudes, Perceptions, and Expectancies in US Youth Receiving Treatment for Mood Disorders and Their Parents.

Supplemental eTable S3. Multinomial and Linear Regression Models Examining Associations Between Marijuana-related Attitudes, Perceptions, Expectancies, and State-Level Cannabis Laws in Total Sample By Respondent Group Status

Supplemental eTable S4. Factors Associated with Intentions to Use Cannabis Among US Youth Receiving Treatment For Mood Disorders, From Ordinal Regression Models, Adjusting for Age, Sex, Caregiver Education, and Site

Supplemental eTable S5. Sensitivity Test: Leave-One-Out Validation (LOOV) Site Analysis Rerunning Multinominal and Linear Regression Models in Subgroup Samples of US Youth Receiving Treatment for Mood Disorders Using *n-1* Samples, Excluding Each Site (n).

Supplemental eTable S6. Sensitivity Test: Subgroup Analysis Rerunning Multinominal and Linear Regression Models in a Subgroup Sample of US Youth Receiving Treatment for Mood Disorders Who Meet Clinical Threshold/Criteria for Current Depression and Their Parents

Supplemental eTable S7. Sensitivity Test: Subgroup Analysis Rerunning Multinominal and Linear Regression Models in US Youth Receiving Treatment for Mood Disorders Who Meet Clinical Threshold/Criteria for Current Anxiety Disorder and Their Parents

Supplemental eTable S8. Sensitivity Test: Subgroup Analysis Rerunning Multinominal and Linear Regression Models in US Youth Receiving Treatment for Mood Disorders Who Report No Lifetime History of Alcohol or Drug Use Problems and Their Parents

Supplemental eTable S9. Sensitivity Test: Subgroup Analysis Rerunning Multinominal and Linear Regression Models in Parent-Child Dyads of US Youth Receiving Treatment for Mood Disorders and Their Parents

Supplemental eFigure S1. Perceptions of mental health benefits and harms related to adolescent cannabis use across frequencies, methods of administration, and chemotypes and related to adolescent CBD use, as a function of respondent group

Supplemental eTable S10. STROBE checklist for article.

----

eMethods S1. MABS survey, development and item details

Survey Description with Item Types and Categories: The MABS Survey is an electronically administered self-report questionnaire w/ branch logic that includes 129 items querying cannabis and CBD related attitudes, perceptions, and behaviors, including acceptability, health perception of risk for harm and medical benefit, expectancies about cannabis and CBDs' effects on mood, anxiety, and cognition, along with parent-youth communication, parenting practices, and demographics and clinical characteristics. Item types and categories are described in detail below:

Sociodemographics (5-items): Demographic information was collected on sex (text response), age in years (text response), state of residence (text response), household composition, parent education level. Parent respondents were also asked to provide information on their relationship with the child receiving mood disorder treatment and the child's age.

Clinical/treatment characteristics (6-items): Respondents were asked if they/their child was currently receiving or had ever received treatment for a mental health condition in their lifetime. Respondents who answered 'yes' to lifetime or current mental health treatment were asked what mental health symptoms/disorders they/their child had received treatment for and were instructed to mark all that apply from a list of 18 different psychiatric symptom categories including depression, anxiety, sleep problems, low motivation/effort, trauma reactivity, attention problems, impulsivity, hyperactivity, drug/alcohol problems, conduct problems (getting into trouble at school or with the police), aggression or violence,

suicidal thoughts or behaviors, non-suicidal self-injurious behaviors (cutting, scratching, burning oneself), paranoia, social difficulties, bipolar disorder or mania, psychotic symptoms/disorders (delusions or hallucinations, and other symptoms (text response). Respondents who answered ‘yes’ to current mental health treatment were asked additional questions about the treatment type (e.g., individual counseling, group therapy, psychiatric medication treatment, family therapy, etc.) and setting/environment (outpatient, community mental health, day hospital or intensive outpatient program, inpatient, school mental health program). Respondents were also asked to rate their degree of satisfaction with their/their child’s current mental health treatment using a 5-point likert scale from 1 for “very satisfied” to 5 for “not at all satisfied”.

Attitudes and beliefs (5-items): Respondents were asked to rate how much they agreed or disagreed with statements about medical cannabis and CBD products being “safe and effective treatments” for health conditions and whether “mental health or medical providers should be recommending or prescribing these products for treatment of mental health conditions.” Attitudes about adults vs. child/adolescent treatment populations were assessed separately. Respondents were also asked to rate how much they approved or disapproved of adolescents using marijuana, tobacco, and alcohol, and using medical marijuana, CBD products, and psychiatric medications to treat mental health conditions. All items used a 5-point likert scale with 1 equating to “strongly disagree/disapprove” and 5 equating to “strongly agree/approve”.

Access/Availability (1-item): All respondents were asked about how easy or difficult it would be for them/their child to obtain cannabis(access) using a 5-point likert scale.

Perceived Drug use norms (1-item): Respondents were asked to estimate how many of their friends smoked tobacco, drank alcohol, used marijuana, used medical cannabis (with a prescription), and used CBD products using a 4-point likert scale with 1 equating to “none” and 5 equating to “all” (of youth’s friends).

Substance use behaviors (4-items): Respondents were asked if they or a household member had used CBD products to treat a mental health condition, and if they or their parent (or their child for parent respondents) had asked their mental health provider about using CBD products to treat mental health conditions in the past-12-months. Parent respondents were asked about both their personal use and child's use CBD products. Respondents who lived in states with MCL were asked similar questions about medical marijuana. Using branch logic, respondents who answered "yes" to questions about personal or household member CBD use were asked 5 additional questions about product type, condition used for, and perceived tolerability and efficacy of CBD.

Substance use intentions (1-item): Respondents were asked to rate the likelihood that they/their child would use tobacco, alcohol, marijuana, medical marijuana, and CBD products in the next 6-months using a 5-point likert scale with 1 equating to "definitely not" and 5 equating to "definitely".

Health perceptions (4-items): Health perceptions for perceived risks for harm and benefits to mental health related to adolescent use of marijuana, tobacco, and alcohol, and adolescent use of medical marijuana, CBD products, psychiatric medications, and psychotherapy/counseling to treat mental health conditions were assessed separately using 4-item likert scales with 1 equating to "no risk/benefit" and 4 equating to "great risk/benefit". Nested within this assessment, respondents were also asked about their perceptions of risk and medical benefit for different cannabis chemotypes (i.e., high THC potency, low THC potency, and high CBD concentrations), product categories (i.e., vaped marijuana, smoked marijuana, edibles, and cannabis concentrates), and use frequencies (i.e., once or twice, occasionally [$\leq$ monthly], or regularly [$\geq$ weekly]).

Cannabis and CBD effect expectancies (48-items total: 16-items each for acute cannabis effects, chronic cannabis effects, and CBD effects): Expectancies for different psychiatric symptom categories, respondents were instructed to answer how they feel that cannabis and CBD affects certain psychiatric symptom clusters in the typical or average user using a 5-point likert scale with 1 equating to "makes much better" and 5 equating to "makes much worse". Expectancies focused on sixteen different

psychiatric symptom clusters and behaviors. In this report we present descriptive results for our main analysis and post-hoc exploratory analyses focused on expectancies related to chronic/regular cannabis use and CBD product use for depressive symptoms, anxiety symptoms/worried thoughts, sleep problems, PTSD symptoms, psychotic symptoms, and suicidal thoughts and behaviors. These psychiatric symptom categories of interest were chosen for our analyses based upon anecdotal data from adult and youth cannabis and CBD users who report using cannabis and CBD to target these symptoms/disorders, data from state-MCL showing that depression, anxiety, PTSD, and insomnia are indicated conditions for medical cannabis treatment, and data from longitudinal/cohort studies showing variation in the likelihood of developing these psychiatric symptoms/disorders as a function of adolescent cannabisuse.[1-3]

Patient and parent perceived knowledge (12-items): Respondents were shown statements about the effects of cannabis and CBD product use on health outcomes, subjective intoxication, overdose risk and the effects of adolescent-onset and adult-onset cannabis use on brain function and mental health outcomes, and were asked to rate how much they agreed or disagreed with these statements.

Parent-child relationships and parenting behaviors (31-items): The survey included items querying parent-child relationship satisfaction (1-item); perceived parental reaction to youth cannabis and other drug use (1-item: separate reactions for alcohol, tobacco, marijuana, medical marijuana, and CBD products); perceived parental support (9-items), parent-child communication about marijuana, and parenting behaviors (20-items; parenting behaviors fall into 2 categories: general and related to youth cannabis use).

Mental health provider practice behaviors and patient-provider communication (11-items): The survey included youth and parent self-report items about healthcare provider screening and practice behaviors related to youth use of cannabis and CBD products, and patient-provider and parent-provider communication/discussions about cannabis and CBD products.

eMethods S2. Measures and Variables Used in Statistical Analysis

Given the large number of survey items, in order to reduce multiple comparisons, we restricted our analysis to a limited number of *a priori* comparisons based upon the main scientific objectives of the current report. The current report had fourfold aims: (1) to characterize medical beliefs, attitudes, expectancies, health and risk perceptions, and use behaviors for CBD and cannabisin the sample; (2) to test for intergenerational differences in these medical beliefs, attitudes, and risk and health perceptions between youth and parent respondents; (3) to examine the influence of state ML on youth and caregiver reported medical beliefs, attitudes, risk and health benefit perceptions, and behaviors; and (4) to identify predictors of youth cannabis and CBD use intentions.

Measures and variables from the MABS survey used for the main analyses of the current report include:

Demographics: A self-report demographic questionnaire was used to assess age (in years), sex (female or male), state of residence, household composition, and highest level of education of the parent of participants. Parent participants were also asked the age and sex and their relationship to the child receiving mental health treatment.

Clinical/Treatment Characteristics: Clinical characteristics were assessed with a question asking “What mental health symptoms have you/your child received treatment for in his/her lifetime?” and included response options for different psychiatric symptom categories (e.g., depression, anxiety disorder, aggression or violent behaviors, eating problems, etc.). Treatment history was assessed with questions asking about lifetime and current mental health treatment receipt and the types of treatments and settings where the child is currently receiving mental health care.

Cannabis and CBD related medical beliefs: Beliefs about cannabis and CBD as medical treatments were assessed by asking participants to report how much they agree with two statements: 1) “Medical marijuana/CBD is a safe and effective treatment for certain mental health condition” and 2) “Mental health or medical providers should be recommending or prescribing medical marijuana/CBD for

treatment of mental health conditions". Parents/caregiver participants were asked about their agreement with these statements for adults (over the age of 18 years) and children and adolescents (under 18 years) separately.

Cannabis and CBD attitudes: Cannabis related attitudes were assessed by asking participants "How do you feel about teenagers (people under 18 years) using marijuana?" with response options of strongly disapprove, somewhat disapprove, neither/nor, somewhat approve, and strongly approve. Response categories were collapsed into disapprove (those who responded strongly or somewhat disapprove), neither, and approve (those who responded strongly or somewhat approve). Medical cannabis and CBD attitudes were calculated by using the same question stem and response options but replacing cannabis in the question with "medical marijuana" and "CBD products".

Cannabis and CBD risk perceptions: Perceived risk of harm related to adolescent cannabis use was assessed by asking participants "How much do you think teenagers (people under 18 years) risk harming themselves (physically or in other ways) if they use cannabis regularly (on a weekly or daily basis)?" with response options of no risk, slight risk, moderate risk, great risk, and don't know. Respondents were classified into perceiving cannabis use as dangerous/risky (those who responded moderate or great risk), not dangerous/risky (those who responded no or slight risk), and uncertain danger/risk (those who responded don't know). Medical cannabis and CBD risk perceptions were calculated in the same way.

Cannabis and CBD health benefit perceptions: Perceived health benefits related to adolescent marijuana, medical marijuana, and CBD use were assessed by asking participants "How beneficial do you think the following things are to teenagers (people under 18 years) for treating mental health conditions and symptoms?" using "marijuana", "medical cannabis obtained from a dispensary", and "cannabidiol or CBD products" as question stems. Response options included no benefit, slight benefit, moderate benefit, great benefit, and don't know. Respondents were classified into perceiving cannabis and CBD use as beneficial (those who responded moderate or great benefit), not beneficial (those who responded no or slight benefit), and uncertain benefit (those who responded don't know).

Cannabis and CBD use intentions: Cannabis and CBD use intentions were assessed by asking participants "In the next six months, how likely is it that you/your child will do the following?" with the following question stems: "use marijuana", "use cannabidiol or CBD products". Response options included definitely not, probably not, possibly, probably, and definitely. Respondents were classified into high use intentions (those who responded definitely or probably), intermediate use intentions (those who responded possibly) and low/no use intentions (those who responded definitely not or probably not).

Cannabis effect expectancies: Cannabis effect expectancies were assessed with the Marijuana Effect Expectancy Questionnaire-Brief (MEEQ-B). [4] The MEEQ-B is a 6-item validated questionnaire that measures individual cannabis expectancies and includes positive effect and negative effect expectancy score. MEEQ-B positive and negative expectancy scores were used as continuous variables in analyses.

Depression: Depression symptom severity was assessed in youth participants with the Patient Health Questionnaire-2-item version Depression scale (PHQ-2).[5] The PHQ-2 is a screener for depression, includes the first two items of the PHQ-9 and has demonstrated validity (Kroenke et al., 2003). PHQ-2 total scores were used a continuous variable of depression symptom severity in analyses.

Anxiety: Anxiety symptom severity was assessed in youth participants with the Generalized Anxiety Disorder-2-item Anxiety scale (GAD-2).[6] The GAD-2 is a screener for anxiety that includes the first two items of the GAD-7. It has good sensitivity and specificity at a cutoff of 3 (Plummer et al., 2016). GAD-2 total scores were used a continuous variable of anxiety symptom severity in analyses.

Supplemental eTable S1. Medical Cannabis Law (MCL) and Recreational Cannabis Law (RCL) Effective Dates by State (A) and Policy Details for each State used in the Sample.

(A) MCL and RCL Effective Dates by State, as of Dec 1, 2023

	MCL		***RCL***	
State	***Legalization Approved***	***Legalization in Effect***	***Legalization Approved***	***Legalization in Effect***
Alaska	11/1998	3/1999	2014	2/2015

Arizona	11/2010	3/2011		
Arkansas	11/2016	11/2016		
California	11/1996	11/1996	2016	11/2016
Colorado	11/2000	6/2001	2012	12/2012
Connecticut	5/2012	10/2012	6/2021	5/2022
Delaware	5/2011	1/2011		
District of Columbia	5/2010	7/2010	2014	2/2015
Florida	11/2016	1/2017		
Hawaii	6/2000	12/2000		
Illinois	8/2013	1/2014	11/2019	1/2020
Louisiana	5/2016	5/2016		
Maine	11/1999	12/1999	2016	1/2017
Maryland	4/2014	6/2014	11/2022	7/2023
Massachusetts	11/2012	1/2013	2016	12/2016
Michigan	11/2008	12/2008	2018	12/2018
Minnesota	5/2014	5/2014	5/2023	8/2023
Missouri	11/2018	12/2018	11/2022	2/2023
Montana	11/2004	11/2004	11/2020	1/2021
Nevada	11/2000	10/2001	2016	1/2017
New Hampshire	7/2013	7/2013		
New Jersey	1/2010	7/2010	11/2020	1/2021
New Mexico	3/2007	7/2007	4/2021	6/2021
New York	7/2014	7/2014	3/2023	3/2023
North Dakota	11/2016	12/2016		
Ohio	6/2016	9/2016	4/2023	12/2023
Oklahoma	6/2018	8/2018		
Oregon	11/1998	12/1998	2014	7/2015
Pennsylvania	4/2016	5/2016		
Rhode Island	1/2006	1/2006	5/2022	5/2022
Utah	11/2018	12/2018		
Vermont	5/2004	7/2004	1/2018	7/2018
Washington	11/1998	11/1998	2012	12/2012
West Virginia	4/2017	7/2019		

Note: States with no cannabis legalization by the end of the study period were excluded from this table.

(B) Description of Cannabis Policy from each US state in the Sample

**State**	**No. of Respondents by State**	**CL Status by State**	**CL Effective Dates by State**	**Details of CL by State***
Colorado (CO)	Youth: 15<br>Parents: 10	RCL and MCL	RCL:<br>Legalization approved	Possession Limits: Individuals may possess up to 2.0 oz. of cannabis flower or concentrates. Individuals may cultivate up

			2012;<br>Legalization in Effect:<br>12/2012<br><br>MCL:<br>Legalization approved<br>11/2000;<br>Legalization in Effect:<br>06/2001	to six cannabis plants with no more than three being mature.<br><br>MCL Qualifying Conditions: Autism spectrum disorder; Cachexia; Cancer; Chronic pain; Chronic nervous system disorders; Glaucoma;<br>HIV or AIDS; Nausea; Persistent Muscle Spasms; Post Traumatic Stress Syndrome; Seizures; Any "condition for which a physician could prescribe an opioid"<br>The health department can approve additional<br>Conditions.
Indiana (IN)	Youth: 13<br>Parents: 19	No MCL or RCL		No MCL or RCL.<br>Low THC: IN has passed a low THC law allowing for the use of cannabis extracts that are high in CBD and low in THC in instances where a physician has recommended such treatment for a patient with a state qualifying condition.
Illinois (IL)	Youth: 10<br>Parents: 12	RCL and MCL*<br><br>*Note IL passed RCL legislation in 11/2019 and legalization went into effect on 01/2020. This was prior to data collection for this site which started on 12/2020 and ended on 06/2021; thus IL was treated as RCL for the purposes of this analysis.	RCL:<br>Legalization approved<br>11/2019*;<br>Legalization in Effect:<br>01/2020*<br>*both prior to survey initiation and completion<br><br>MCL:<br>Legalization approved<br>08/2013;<br>Legalization in Effect:<br>01/2014	New RCL: Age: 21+ Possession Limits: Individuals may possess up to 1.0 oz (30 grams) of cannabis and 500 milligrams of THC contained in cannabis-infused products for state residents (15 g/250mg for non-residents and/or up to 5 grams of cannabis concentrates (2.5 grams for non-residents)<br><br>Possession Limits: Individuals may possess up to 2.0 oz. of cannabis flower or concentrates. Individuals may cultivate up to six cannabis plants with no more than three being mature.<br><br>MCL Qualifying Conditions: Alzheimer's disease; ALS; Anorexia nervosa; "Any medical condition for which an opioid has been or could be prescribed by a physician based on generally accepted standards of care"; Arnold Chiari malformation; Autism; Cachexia/wasting syndrome; Cancer; Causalgia; Chronic Inflammatory Demyelinating Polyneuropathy;<br>Chronic pain; Complex regional pain syndrome type 2; Crohn's Disease; Dystonia; Ehlers-Danlos syndrome; Fibromyalgia; Fibrous dysplasia; Glaucoma; Hepatitis C; HIV/AIDS

				Hydrocephalus; Hydromyelia; Interstitial Cystitis; Irritable bowel syndrome; Lupus Migraines; Multiple Sclerosis; Muscular Dystrophy; Myasthenia Gravis; Myoclonus; Nail patella syndrome; Neuro-Behcet's autoimmune disease; Neurofibromatosis<br>Neuropathy; Osteoarthritis; Parkinson's disease<br>Polycystic kidney disease; PTSD;<br>Reflex Sympathetic Dystrophy (RSD); Rheumatoid Arthritis; Sjogren's syndrome; Spinal cord disease; Spinocerebellar Ataxia (SCA); Superior canal dehiscence syndrome<br>Syringomyelia; Tarlov cysts; Tourette's syndrome; Traumatic brain injury and post-concussion syndrome; and Ulcerative colitis.<br>The health department can approve additional<br>Conditions.
Maryland (MD)	Youth: 17<br>Parents: 9	RCL and MCL*<br><br>*Note MD passed RCL legislation in 11/2022, but this was after data collection for this site which ended on 07/2021; thus, MD was treated as MCL for the purposes of this analysis.	RCL: Legalization approved 11/2022*; Legalization in Effect: 07/2023*<br>*both after survey completion<br><br>MCL: Legalization approved 04//2014; Legalization in Effect: 06/2014	New RCL: Possession Limits: Individuals may possess up to 1.5 oz. of cannabis and/or 12 grams of cannabis concentrates and will be permitted to cultivate up to two cannabis plants for personal use.<br><br>MCL: Possession Limits: Patient may receive up to 120 gm of cannabis flower or 36 gm of THC via an infused product every 30 days. Patients may cultivate up to four cannabis plants per residence. Patient may possess no more than 4 oz. of cannabis at one time.<br><br>MCL Qualifying Conditions: Cachexia; Anorexia, or Wasting Syndrome; Chronic or severe pain; Glaucoma; PTSD; Seizures; Severe or persistent muscle spasms; Severe nausea; Seizures; and any other severe condition "for which other medical treatments have been ineffective … if the symptoms reasonably can be expected to be relieved by" cannabis
Michigan (MI)	Youth: 10<br>Parents: 6	RCL and MCL	RCL Legalization approved 2018; Legalization in	Possession Limits: Individuals ages 21 years and older may possess up to 2.5 oz of marijuana; up to 15 gm may be concentrates. Within a residence, an adult may cultivate up to twelve cannabis plants for personal use and possess up to 10 oz of

			Effect: 12/2018<br><br>MCL: Legalization approved 11/2016; Legalization in Effect: 01/2017	any cannabis produced by cannabis cultivated on the premises.<br><br>MCL Qualifying Conditions:<br>Cancer, HIV/AIDS, hepatitis C, ALS, Crohn's disease, nail patella, glaucoma, autism, Alzheimer's, PTSD, arthritis, inflammatory bowel disease, OCD, Parkinson's, rheumatoid arthritis, spinal cord injury, cerebral palsy, colitis, ulcerative colitis, Tourette's syndrome, chronic pain, cachexia, severe nausea, seizures, or severe and persistent muscle spasms.<br><br>The department of health can add conditions.
Minnesota (MN)	Youth: 13<br>Parents: 7	RCL and MCL*<br><br>*Note MN passed RCL legislation in 05/2023, but this was after data collection for this site which ended on 05/2021; thus, MN was treated as MCL for the purposes of this analysis.	RCL: Legalization approved 05/2023*; Legalization in Effect: 08/2023*<br>*both after survey completion<br><br>MCL: Legalization approved 05/2014; Legalization in Effect: 05/2013	New RCL: Possession Limits: Individuals may possess up to 1.5 oz. of cannabis and/or 12 grams of cannabis concentrates and will be permitted to cultivate up to two cannabis plants for personal use.<br><br>Possession Limits: A 30-day supply of non-inhaled forms of cannabis as determined by the pharmacist dispensing the cannabis. Beginning March 1, 2022, patients are permitted to access herbal forms of cannabis, and may purchase up to 2.5 oz. of medical cannabis flower per 14-day period from licensed providers.<br><br>MCL Qualifying Conditions:<br>Age-related macular degeneration; Alzheimer's disease; ALS; Autism; Cancer/cachexia; Chronic pain; Chronic vocal or motor tic disorder *; Crohn's disease; Glaucoma; HIV/AIDS; Intractable pain<br>PTSD; Seizures; Severe and persistent muscle spasms; Sickle cell disease *; Sleep apnea; Terminal illness; Tourette's Syndrome<br>* Effective August 2021<br>The department may add conditions, provided the legislature does not object.
Pennsylvania (PA)	Youth: 2<br>Parents: 2	MCL	MCL: Legalization passed on 04/2016;	Possession Limits: 90-day supply — As initially enacted, only cannabis-infused pills, oils, topical ointments, tinctures or liquids were allowed. On April 16, 2018,

			Legalization in Effect on 05/2016	the state Health Secretary approved an advisory board recommendation to permit dispensaries to also begin providing herbal cannabis. Under the rule change, patients are permitted to vaporize herbal cannabis, but are still prohibited under the law from smoking.<br><br>MCL Qualifying Conditions:<br>ALS (Lou Gehrig's disease); Anxiety disorders; Autism; Cancer, including remission therapy; Chronic Hepatitis C; Crohn's disease; Damage to the nervous tissue of the central nervous system (brain-spinal cord) with objective neurological indication of intractable spasticity and other associated neuropathies; Dyskinetic/spastic movement disorders; Epilepsy; Glaucoma; HIV/AIDS; Huntington's disease; Inflammatory bowel disease; Intractable seizures; Multiple Sclerosis; Opioid dependency; Neurodegenerative disorders; Neuropathies; Parkinson's disease; PTSD; Sickle cell anemia; Severe chronic or intractable pain; Terminal illness (defined as 12 months or fewer to live); Tourette syndrome:<br>Other conditions that are recommended by the advisory board and approved by the secretary
Wisconsin (WI)	Youth: 1<br>Parents: 0	No MCL or RCL		No MCL or RCL.<br>Low THC: WI has passed a low THC law allowing for the use of cannabis extracts that are high in CBD and low in THC in instances where a physician has recommended such treatment for a patient with a state qualifying condition.
Idaho (ID)	Youth: 1<br>Parents: 1	No MCL or RCL		No MCL or RCL.
Texas (TX)	Youth: 1<br>Parents: 0	No MCL or RCL		No MCL or RCL.<br>Low THC: WI has passed a low THC law allowing for the use of cannabis extracts that are high in CBD and low in THC in instances where a physician has recommended such treatment for a patient with a state qualifying condition.
New York (NY)	Youth: 1<br>Parents: 0	RCL and MCL*	RCL Legalization approved	New RCL: Possession Limits: Individuals ages 21 and older may possess up to 3.0 oz. of cannabis flower and/or 24 grams of

		*Note NY passed RCL legislation in 3/2023 but this was after data collection for this participant on 4/17/22, thus NY was treated as MCL for the analysis.	3/2023*; Legalization in Effect: 3/2023* (with planned effective initiation date of retail sales set for 6/2024. *both after survey completion<br><br>MCL: Legalization approved 7/2014; Legalization in Effect: 7/2014	cannabis concentrates and will be permitted to cultivate up to six cannabis plants (3 mature and 3 immature) for personal use.<br><br>MCL: Possession Limits: Patient may purchase up to a 60-day supply of cannabis products for medical use. Patients may cultivate up to six cannabis plants (3 mature) per private residence and may possess a total of up to 5 pounds of cannabis from their harvest.<br><br>MCL Qualifying Conditions: Cancer, HIV/AIDS, ALS, Parkinson's, MS, spinal cord injury with spasticity, neuropathy, epilepsy, inflammatory bowel disease, PTSD, Huntington's, some types of pain, or substance use disorder. The above conditions must also be accompanied by wasting, severe or chronic pain, severe nausea, seizures, or severe or persistent spasms, PTSD, or opioid use disorder.

Notes: No states that youth patients and parent respondents reported living in changed their state-level ML status during the data collection period.

Abbreviations: ALS = Amyotrophic Lateral Sclerosis. MCL = Medical Cannabis Legalization – defined as a state that has passed and effected medical cannabis law at the time of the survey administration. RCL = Recreational Cannabis Legalization – defined as a state that has passed and effected recreational cannabis law at the time of the survey administration. All states that passed RCL had previously passed MCL. No-CL = No Cannabis Legalization – defined as a state with no medical or recreational cannabis legalization at the time of the survey administration. OCD = Obsessive Compulsive Disorder. Post-traumatic Stress Disorder = PTSD. THC = delta-9-tetrahydrocannabinol – the main psychoactive ingredient in the cannabis plant. Column 4 shows MCL and RCL Effective Dates by State, as of March 2022. For states with no cannabis legalization at the time of survey administration columns 4 and 5 are left blank in this table. *Data obtained from: 1. NORCL (2022). State Laws. National Organization for Cannabis Legalization (NORCL) online information on state cannabis laws. Accessed on 5/7/23: https://noRCL.org/laws/ 2. MPP (2022). Key Aspects of State and D.C. Medical Cannabis Laws. Cannabis Policy Project online published report, published May 16, 2022. Accessed on 1/12/23: https://www.mpp.org/assets/pdf/issues/medical-marijuana/State-and-DC-MMJ-Laws.pdf

Supplemental eResults S1. Description of Neutral and Uncertain Response Outcomes from Main analyses

Regarding analyses focused on uncertain (e.g., "I don't know") or neutral response outcomes (e.g., "neither approve nor disapprove" vs. "approve" or "disapprove"), some respondent group, state-level ML status, and respondent group-by-state ML status differences were observed (see

Table 2, eTables S2-S3 and eFigure S1 C and D). As shown in Table 2, youth were more likely than parents to report neutral attitudes ("neither/nor" vs. "disagree") about youth marijuana, medical marijuana, and CBD use. Focusing on perceptions: As shown in eFigure S1C-D, the cannabinoid products that both youth and parent respondents were most likely to report "I don't know" when asked about perceived benefits and harms related to teen use were medical cannabis (obtained from a dispensary) and CBD products, respectively. Youth respondents, in general, were more likely than parent respondents to report uncertainty (i.e., responding "I don't know") related to perceived health benefits and risks from adolescent use of CBD products and cannabis across methods of administration and chemotypes. A subgroup of both youth and parent respondents reported uncertainty (i.e., responding "I don't know") related to perceived risks for harm from adolescent use of cannabis and CBD products, with respondent group differences reaching statistical significance only for perceived health benefits related to youth CBD use (% of youth vs. parents reporting "I don't know": 14.9% vs. 1.6%, AOR = 19.54 (2.33, 162.320, $p<0.001$). Two other notable points. First, parent respondents only had a higher proportion of respondents endorsing "I don't know" compared to youth respondents on two items – perception of risk for harm related to teen medical cannabis use (from a dispensary) and CBD product use, although these comparisons did not achieve statistical significance. For all other risk perception items and all health benefit perception items, youth were more likely to report "I don't know" compared to parents. Second, a higher proportion of parent respondents reported uncertainty regarding risk perceptions related to different cannabis chemotypes (cannabis with high THC, low THC, high CBD concentrations) when compared to uncertain responses regarding risk perception related to frequency and health benefit perceptions in general.

While we did identify differences as a function of state-level ML status, the directionality of these relationships was more variable and did not always fit the same pattern of positive responses (i.e., RCL > MCL > No-CL). This suggests that these relationships are more complicated and that the role of certainty vs uncertainty of knowledge may be important in decision-making related to future cannabis and CBD use, and may vary in complex ways based upon state-level ML status. One observation from our state-level ML analysis focused on neutral/uncertain response outcomes from Table 2: all significant neutral/uncertain response results were focused questions querying medical cannabis and CBD-related medical beliefs and risk and health benefit perceptions. Taken together, these results suggest that youth and parents may be less confident and more uncertain about their knowledge with regard to CBD and medical cannabis compared to recreational cannabis use.

eTable S2. Multinominal and Linear Regression Models Examining Marijuana-related Attitudes, Perceptions, and Expectancies Using Respondent Group and State-Level Cannabis Law Status As Between-Subject Factors, Covarying for Sex and Parent Education.

	**Group comparison:** Main Effect	**State CL Status comparison**: Main Effect			**Group-By-State-CL-Status Comparison:** Interaction Effect		
					Youth vs. Parents		
	Youth vs. Parents	MCL vs. No-CL	No-CL vs. RCL	MCL vs. RCL	MCL vs. No-CL	No-CL vs. RCL	MCL vs. RCL
	Wald Chi-Squared Test Statistic	Wald Chi-Squared Test Statistic	Wald Chi-Squared Test Statistic	Wald Chi-Squared Test Statistic	Wald Chi-Squared Test Statistic	Wald Chi-Squared Test Statistic	Wald Chi-Squared Test Statistic
**MC is a safe & effective txt**							
Agree	0.83	4.22*	1.80	2.42	0.80	0.95	0.00
Neither/nor	2.35	183.35***	186.69***	3.00±	--[X]	--[X]	1.93
Disagree	Ref.	Ref.	Ref.	Ref.	Ref.	Ref.	Ref.
**CBD is a safe & effective txt**							
Agree	0.11	1.50	1.02	0.24	0.04	0.83	0.92
Neither/nor	0.05	2.92±	0.73	0.58	0.01	0.72	0.67
Disagree	Ref.	Ref.	Ref.	Ref.	Ref.	Ref.	Ref.
**Attitude about regular cannabis use by youth**							
Approve	320.41***	--[X]	0.06	876.15***	--[X]	--[X]	--[X]
Neither/nor	5.05*	0.06	0.08	0.03	0.60	0.37	0.02
Disapprove	Ref.	Ref.	Ref.	Ref.	Ref.	Ref.	Ref.
**Attitude about MC use by youth**							
Approve	7.47*	0.23	0.92	2.63	0.52	0.07	0.97
Neither/nor	3.50±	0.00	0.16	0.39	0.08	0.05	0.12
Disapprove	Ref.	Ref.	Ref.	Ref.	Ref.	Ref.	Ref.
**Attitude about CBD use by youth**							
Approve	2.00	0.00	1.20	5.25*	2.02	0.27	2.27
Neither/nor	0.56	0.08	1.22	2.05	0.31	0.06	0.80
Disapprove	Ref.	Ref.	Ref.	Ref.	Ref.	Ref.	Ref.

**Perceived risk for harm related to regular cannabis use by youth**							
Mod/high risk	4.40*	0.37	0.61	0.20	0.28	1.33	0.70
Do not know	1.18	0.01	0.14	208.38***	0.00	0.49	--[X]
No/low risk	Ref.	Ref.	Ref.	Ref.	Ref.	Ref.	Ref.
**Perceived risk for harm related to MC use by youth**							
Mod/high risk	5.85*	0.01	0.01	0.06	0.56	0.44	0.04
Do not know	374.19***	0.00	181.61***	452.93***	0.06	162.35***	--[X]
No/low risk	Ref.	Ref.	Ref.	Ref.	Ref.	Ref.	Ref.
**Perceived risk for harm related to CBD use by youth**							
Mod/high risk	0.81	0.08	0.26	1.27	0.42	0.24	0.13
Do not know	341.45***	0.02	144.94***	278.33***	0.16	146.75***	--[X]
No/low risk	Ref.	Ref.	Ref.	Ref.	Ref.	Ref.	Ref.
**Perceived benefit for mental health related to regular cannabis use by youth**							
Mod/high benefit	6.06*	0.71	0.01	0.65	0.57	0.25	0.17
Do not know	--[X]	--[X]	--[X]	0.14	--[X]	--[X]	--[X]
No/low benefit	Ref.	Ref.	Ref.	Ref.	Ref.	Ref.	Ref.
**Perceived benefit for mental health related to MC use by youth**							
Mod/high benefit	10.12**	0.00	0.10	1.62	0.02	0.55	0.12
Do not know	--[X]	--[X]	--[X]	0.31	--[X]	--[X]	--[X]
No/low benefit	Ref.	Ref.	Ref.	Ref.	Ref.	Ref.	Ref.
**Perceived benefit for mental health related to CBD use by youth**							
Mod/high benefit	1.46	1.00	1.87	1.78	0.64	0.23	0.28
Do not know	270.62***	213.80***	--[X]	462.84***	--[X]	--[X]	--[X]

No/low benefit	Ref.	Ref.	Ref.	Ref.	Ref.	Ref.	Ref.
**Expectancies**	**Group Comparison**: Youth vs. Parent	**State CL Status Comparison**: No-CL (1) vs. MCL (2) vs. RCL (3)					
	T-test Statistic	T-test Statistic					
MEEQ-B positive exp.	-0.70	0.39					
MEEQ-B negative exp.	1.75	0.51					

Note: Multinomial logistic regression models used medical beliefs, attitudes, risk perceptions, health benefit perceptions as outcome variables (reference group for each variable shown above) with respondent group membership and state-level ML status as between subject factor covarying for sex and caregiver education. Linear regression models used MEEQ-B positive and negative expectancy scores as outcome variables with respondent group membership and state-level ML status as between-subjects factor covarying for sex and caregiver education. Results from the multinomial logistic regression models are presented as Wald Chi-Squared Test statistics. Results from the linear regression models are presented as t-test statistics. Statistical significance is shown with ± for $p < 0.10$, * for $p < 0.05$, ** for $p < 0.01$, and *** for $p < 0.001$. [X]Comparisons that had dependent variable levels in one of the comparator subpopulations with zero frequencies. Examples include the following: Among respondents living in states with RCL (i.e., RCL group), 0.0% responded with "don't know" to the question about perceived risk for harm related to CBD use in youth. Among parent respondents, 0.0% responded with "do not know" to question about perceived mental health benefits from regular (weekly or more) cannabis use by youth. Among respondents living in states without ML (i.e., No-CL group), 0.0% responded with "do not know" to question about perceived mental health benefits from regular cannabis use in youth. Among parent respondents, 0.0% responded with "do not know" to question about perceived mental health benefits from medical cannabis treatment for youth in youth. Abbreviations: CBD: cannabidiol; MM: medical marijuana; MJ: marijuana; No-CL: No cannabis laws; MCL: Medical Cannabis Laws; RCL: Recreational Cannabis Law; Marijuana-related medical beliefs: Cannabis is a safe and effective treatment for pediatric mental health conditions; CBD-related medical beliefs: CBD is a safe and effective treatment for pediatric mental health conditions; MEEQ-B positive exp: MEEQ-B; negative exp:

eTable S3. Main and Interaction Effect Results from Multinomial and Linear Regression Models Examining Associations Between Marijuana-related Attitudes, Perceptions, Expectancies, and State-Level Cannabis Laws, by Respondent Group Status

	**State CL status analysis: Youth Respondents[A]**			**State CL status analysis: Parent Respondents[A]**			**Group-by-state-CL status interaction analysis: Youth vs. Parents[B]**		
	MCL vs. No-CL	No-CL vs. RCL	MCL vs. RCL	MCL vs. No-CL	No-CL vs. RCL	MCL vs. RCL	MCL vs. No-CL	No-CL vs. RCL	MCL vs. RCL
	AOR (95% CI)	*AOR (95% CI)*	*AOR (95% CI)*	*AOR (95% CI)*	*AOR (95% CI)*	*AOR (95% CI)*	*AOR (95% CI)*	*AOR (95% CI)*	*AOR (95% CI)*
**MC is a safe & effective txt**									
Agree	2.91 (0.15, 56.10)	2.27 (0.19, 26.82)	2.29 (0.28, 18.54)	7.35 (1.34, 40.29)*	0.23 (0.05, 1.02)±	1.16 (0.18, 7.42)	0.26 (0.01, 4.93)	3.86 (0.26, 58.17)	1.14 (0.09, 15.09)
Neither/nor	1.14 (0.08, 15.72)	0.83 (0.05, 13.69)	1.50 (0.15, 15.29)	--[X]	--[X]	7.43 (0.64, 86.37)	--[X]	--[X]	0.10 (0.00, 2.53)
Disagree	Ref.	Ref.	Ref.	Ref.	Ref.	Ref.	Ref.	Ref.	Ref.

**CBD is a safe & effective txt**									
Agree	2.18 (0.12, 41.16)	1.28 (0.16, 19.72)	3.06 (0.25, 36.94)	3.53 (0.49, 25.19)	0.28 (0.05, 1.73)	0.41 (0.05, 3.31)	0.70 (0.02, 21.18)	3.87 (0.21, 71.12)	4.43 (0.21, 93.57)
Neither/nor	6.33 (0.25, 163.62)	2.00 (0.11, 36.61)	12.04 (0.74, 195.12)±	8.27 (0.73, 93.23)±	0.34 (0.03, 3.87)	2.72 (0.25, 30.23)	0.79 (0.02, 42.04)	5.00 (0.12, 207.62)	4.23 (0.13, 134.80)
Disagree	Ref.	Ref.	Ref.	Ref.	Ref.	Ref.	Ref.	Ref.	Ref.
**Attitude about youth regular cannabis use**									
Approve	0.33 (0.06, 1.64)	0.69 (0.14, 3.29)	0.26 (0.06, 1.12)±	--[X]	--[X]	--[X]	--[X]	--[X]	--[X]
Neither/nor	2.20 (0.21, 23.13)	0.22 (0.02, 2.45)	0.52 (0.12, 2.28)	0.91 (0.05, 18.04)	0.60 (0.04, 9.92)	0.80 (0.06, 11.22)	4.21 (0.11, 159.27)	0.35 (0.01, 10.35)	1.20 (0.07, 20.07)
Disapprove	Ref.	Ref.	Ref.	Ref.	Ref.	Ref.	Ref.	Ref.	Ref.
**Attitude about youth MC use**									
Approve	2.13 (0.35, 13.01)	0.33 (0.05, 2.39)	0.45 (0.07, 3.04)	1.82 (0.41, 8.07)	0.20 (0.04, 1.09)±	0.15 (0.02, 0.90)*	2.22 (0.26, 19.26)	0.74 (0.08, 7.06)	3.04 (0.33, 27.87)
Neither/nor	0.96 (0.13, 6.92)	0.49 (0.06, 3.91)	0.19 (0.02, 1.70)	3.31 (0.45, 24.08)	0.42 (0.08, 2.38)	0.49 (0.08, 3.70)	1.43 (0.12, 16.94)	0.76 (0.06, 9.36)	0.64 (0.05, 8.00)
Disapprove	Ref.	Ref.	Ref.	Ref.	Ref.	Ref.	Ref.	Ref.	Ref.
**Attitude about CBD use by youth**									
Approve	5.61 (0.67, 47.23)	0.18 (0.02, 1.68)	0.78 (0.07, 8.14)	1.39 (0.35, 5.60)	0.20 (0.04, 1.05)±	0.09 (0.02, 0.54)**	5.75 (0.51, 64.36)	0.52 (0.04, 6.14)	7.21 (0.5, 94.17)
Neither/nor	2.59 (0.29, 32.98)	0.77 (0.07, 9.17)	0.44 (0.05, 4.24)	2.10 (0.37, 11.99)	0.13 (0.01, 1.17)	0.27 (0.04, 1.76)	2.12 (0.15, 29.55)	1.39 (0.09, 20.82)	3.50 (0.22, 54.83)
Disapprove	Ref.	Ref.	Ref.	Ref.	Ref.	Ref.	Ref.	Ref.	Ref.
**Perceived risk related to youth regular cannabis use**									
Mod/high risk	1.29 (0.28, 5.94)	1.90 (0.40, 8.92)	3.29 (0.87, 12.51)±	1.47 (0.29, 7.45)	0.68 (0.11, 4.24)	0.93 (0.14, 6.08)	0.58 (0.07, 4.48)	3.51 (0.42, 29.63)	2.38 (0.31, 18.24)
Do not know	2.51 (0.15, 43.00)	4.33 (0.16, 121.10)	26.36 (1.11, 624.24)*	1.39 (0.05, 39.46)	0.06 (0.00, 9.69)	--[X]	0.92 (0.02, 53.19)	9.86 (0.06, 400.46)	--[X]
No/low risk	Ref.	Ref.	Ref.	Ref.	Ref.	Ref.	Ref.	Ref.	Ref.
**Perceived risk related to youth MC use**									
Mod/high risk	0.52 (0.11, 2.38)	1.90 (0.40, 8.86)	1.05 (0.24, 4.58)	0.74 (0.20, 2.67)	1.60 (0.42, 6.11)	1.32 (0.34, 5.16)	0.98 (0.07, 3.23)	1.93 (0.28, 13.35)	0.83 (0.13, 5.36)
Do not know	1.14 (0.17, 7.85)	2.69 (0.29, 24.68)	3.67 (0.47, 28.56)	0.94 (0.11, 7.93)	2.72E-9 (3.00E-8, 2.47E-10)***	--[X]	0.72 (0.05, 10.87)	1.11E-8 (6.68E-10 , 1.87E-7)***	--[X]
No/low risk	Ref.	Ref.	Ref.	Ref.	Ref.	Ref.	Ref.	Ref.	Ref.

**Perceived risk related to youth CBD use**									
Mod/high risk	0.64 (0.13, 3.31)	2.39 (0.39, 14.64)	1.51 (0.30, 7.74)	0.94 (0.22, 4.13)	2.42 (0.48, 12.22)	2.89 (0.62, 13.43)	0.50 (0.06, 4.16)	1.78 (0.18, 18.00)	0.67 (0.08, 5.59)
Do not know	1.06 (0.13, 8.40)	5.61 (0.37, 84.24)	5.43 (0.38, 77.46)	1.08 (0.14, 8.62)	--[X]	--[X]	0.57 (0.04, 8.94)	1.89E-8 (1.06E-9, 2.26E-7)***	--[X]
No/low risk	Ref.	Ref.	Ref.	Ref.	Ref.	Ref.	Ref.	Ref.	Ref.
**Perceived health benefit related to youth regular cannabis use**									
Mod/high benefit	1.03 (0.29, 3.73)	0.64 (0.17, 2.35)	0.59 (0.19, 1.85)	0.46 (0.07, 3.32)	0.97 (0.14, 6.88)	0.32 (0.03, 3.85)	2.42 (0.24, 24.17)	0.57 (0.07, 5.05)	1.72 (0.13, 23.26)
Do not know	--[X]	--[X]	1.20 (0.25, 5.66)	--[X]	--[X]	--[X]	--[X]	--[X]	--[X]
No/low benefit	Ref.	Ref.	Ref.	Ref.	Ref.	Ref.	Ref.	Ref.	Ref.
**Perceived health benefit related to youth MC use**									
Mod/high benefit	0.83 (0.23, 3.06)	0.39 (0.09, 1.59)	0.21 (0.06, 0.76)*	1.00 (0.28, 3.53)	0.74 (0.19, 2.89)	0.34 (0.07, 1.57)	0.87 (0.15, 5.15)	0.49 (0.09, 3.18)	0.71 (0.11, 4.78)
Do not know	5.35 (0.52, 54.95)	0.23 (0.02, 2.84)	1.07 (0.20, 5.73)	--[X]	--[X]	--[X]	--[X]	--[X]	--[X]
No/low benefit	Ref.	Ref.	Ref.	Ref.	Ref.	Ref.	Ref.	Ref.	Ref.
**Perceived health benefit related to youth CBD use**									
Mod/high benefit	0.71 (0.17, 2.95)	0.70 (0.15, 2.98)	0.46 (0.13, 1.70)	2.25 (0.41, 12.39)	0.45 (0.08, 2.46)	1.00 (0.25, 40.06)	0.47 (0.07, 3.01)	1.60 (0.24, 10.81)	1.62 (0.27, 9.92)
Do not know	3.77 (0.36, 39.12)	0.33 (0.03, 3.78)	1.00 (0.20, 5.10)	--[X]	--[X]	--[X]	--[X]	--[X]	--[X]
No/low benefit	Ref.	Ref.	Ref.	Ref.	Ref.	Ref.	Ref.	Ref.	Ref.
**Cannabis Expectancies[C]**	**State CL status: Youth Respondents:** State CL Status associations (No-CL (1) vs. MCL (2) vs. RCL (3))			**State CL status: Parent Respondents:** State CL Status associations (No-CL (1) vs. MCL (2) vs. RCL (3))			**Group-by-State-CL status interaction: Youth vs Parent respondents**: State CL Status associations (No-CL (1) vs. MCL (2) vs. RCL (3))		
	Beta (95% CI)			*Beta (95% CI)*			*Beta (95% CI)*		
MEEQ-B positive exp.	-0.02 (-0.25, 0.21)			0.10 (-0.13, 0.28)			0.03 (-0.13, 0.19)		
MEEQ-B negative exp.	0.11 (-0.11, 0.34)			-0.10 (-0.32, 0.15)			0.04 (-0.12, 0.20)		

Note: [A]Multinomial logistic regression models stratified by respondent group (youth and parent results presented separately) included medical beliefs, attitudes, risk perceptions, health benefit perceptions as outcome variables (reference group for each variable shown above) with state-level ML status as between subjects factor covarying for age, sex, and caregiver education for ML analyses. [B]Group-by-State-ML-status interaction analysis presented in the last column is from multinomial logistic regression models that used medical beliefs, attitudes, risk perceptions, health benefit perceptions as outcome variables (reference group for each variable shown above) with respondent group membership and state-level ML status as between subject factor covarying for sex and caregiver education. [C]Linear regression models included MEEQ-B positive and negative expectancy scores as outcome variables with respondent group membership as between-subjects factor covarying for sex, caregiver education, and site for respondent group analyses and with state-level ML status as between-subjects factor covarying for age, sex, and caregiver education for ML analyses. Results from the multivariate logistic regression models are presented as AOR with 95% CI with statistical significance shown with ± for $p < 0.10$, * for $p < 0.05$, ** for $p < 0.01$, and *** for $p < 0.001$. Results from the linear regression models are presented as non-standardized β parameter estimates with 95% CI with statistical significance shown with ± for $p < 0.10$, * for $p < 0.05$, ** for $p < 0.01$, and *** for $p < 0.001$. [X]Comparison had dependent variable levels in one of the comparator subpopulations with zero frequencies. Abbreviations: CBD: cannabidiol; MM: medical marijuana; MJ: marijuana; No-CL: No cannabis laws; MCL: Medical Cannabis Laws; RCL: Recreational Cannabis Law; Marijuana-related medical beliefs: Cannabis is a safe and effective treatment for pediatric mental health conditions; CBD-related medical beliefs: CBD is a safe and effective treatment for pediatric mental health conditions; MEEQ-B positive exp: MEEQ-B; negative exp:

eTable S4. Factors Associated with Intentions to Use Cannabis Among US Youth Receiving Treatment For Mood Disorders, From Ordinal Regression Models, Adjusting for Age, Sex, Parent Education, and Site

Factor	Grouping Variable: Intention to Use Marijuana				
	Participants responding "Definitely" or "Probably" (n=23)	Participants responding "Possibly" (n=7)	Participants responding "Definitely Not" or "Probably Not" (n=54)	Parameter Estimate (95% CI)	P-Value
**Mean Age**	18.39 [2.89]	19.86 [3.18]	16.89 [3.31]	0.14 (0.00, 0.28)	*0.051*
**Sex**					
Female	14 (60.9%)	7 (100.0%)	35 (66.0%)	0.08 (-0.95, 1.10)	*0.89*
Male	9 (39.1%)	0 (0.0%)	18 (34.0%)	Ref.	
**Lifetime Mental Health Symptom Categories**					
Depression	22 (95.7%)	6 (85.7%)	40 (74.1%)	1.61 (-0.04, 3.26)	*0.055*
Anxiety/worry	22 (95.7%)	6 (85.7%)	47 (87.0%)	0.99 (-0.82, 2.80)	*0.28*
Anger/irritability	7 (30.4%)	0 (0.0%)	17 (31.5%)	0.12 (-0.95, 1.18)	*0.83*
Amotivation/anhedonia	12 (52.2%)	2 (28.6%)	26 (48.1%)	0.10 (-0.88, 1.09)	*0.84*
Sleep problems	9 (39.1%)	3 (42.9%)	26 (48.1%)	-0.55 (-1.53, 0.43)	*0.27*
Social difficulties	9 (39.1%)	2 (28.6%)	12 (22.2%)	1.04 (-0.01, 2.09)	*0.053*
Aggression	4 (17.4%)	0 (0.0%)	2 (3.7%)	1.68 (-0.09, 3.44)	*0.06*
Impulsivity	6 (26.1%)	2 (28.6%)	8 (13.1%)	1.41 (0.19, 2.62)	*0.02*

Hyperactivity	4 (17.4%)	1 (14.3%)	10 (18.5%)	0.37 (-0.88, 1.62)	*0.57*
Inattention	8 (34.8%)	1 (14.3%)	17 (31.5%)	0.26 (-0.75, 1.27)	*0.61*
PTSD/Trauma reactivity	10 (43.5%)	4 (57.1%)	8 (14.8%)	1.42 (0.31, 2.53)	*0.012*
Alcohol or drug use problems	5 (21.7%)	2 (28.6%)	2 (3.7%)	1.18 (-0.32, 2.69)	*0.12*
Eating disorder behaviors	8 (34.8%)	3 (42.9%)	10 (18.5%)	1.18 (0.06, 2.31)	*0.039*
Bipolar disorder or mania	7 (30.4%)	2 (28.6%)	8 (14.8%)	0.46 (-0.77, 1.69)	*0.46*
Psychosis, delusions, hallucinations	5 (21.7%)	1 (14.3%)	4 (7.4%)	0.84 (-0.59, 2.27)	*0.25*
Non-suicidal self-injurious thoughts and behaviors	13 (56.5%)	6 (85.7%)	17 (31.5%)	1.22 (0.16, 2.28)	*0.02*
Suicidal thoughts and behaviors	15 (65.2%)	3 (42.9%)	20 (37.0%)	1.42 (0.31, 2.53)	*0.01*
**Total Psychiatric Problems score[A]**	7.22 [3.25]	6.29 [3.04]	5.04 [3.48]	0.20 (0.05, 0.34)	*0.007*
**PHQ-2 depression total score**	2.65 [2.01]	2.57 [1.13]	2.17 [1.88]	0.09 (-0.16, 0.35)	*0.48*
**GAD-2 anxiety total score**	3.35 [1.94]	2.57 [1.81]	2.54 [2.00]	0.16 (-0.08, 0.40)	*0.20*
**MEEQ-B positive exp. score**	4.29 [0.55]	3.95 [0.36]	3.48 [0.83]	1.78 (0.87, 2.69)	*<0.001*
**MEEQ-B negative exp. score**	3.17 [0.54]	3.81 [0.42]	3.31 [0.84]	-0.80 (-0.71, 0.55)	*0.80*
**Past Year CBD use by youth[X]**					
Yes	5 (22.7%)	1 (14.3%)	5 (11.4%)	0.82 (-0.48, 2.12)	*0.22*
No	17 (77.3%)	6 (85.7%)	39 (88.6%)	Ref.	
**Past Year CBD use by household member**					
Yes	6 (27.3%)	2 (28.6%)	8 (18.2%)	0.80 (-0.36, 1.96)	*0.18*
No	16 (72.7%)	5 (71.4%)	36 (81.8%)	Ref.	
**Past Year MC use by household member[B]**					
Yes	2 (10.5%)	0 (0.0%)	3 (8.6%)	-0.59 (-2.49, 1.31)	*0.54*
No	19 (89.5%)	7 (100.0%)	32 (91.4%)	Ref.	
**MC is a safe & effective txt**					
Agree	22 (95.7%)	6 (85.7%)	35 (66.0%)	1.57 (-0.68, 3.83)	*0.17*
Neither/nor	0 (0.0%)	1 (14.3%)	13 (24.5%)	-0.79 (-3.82, 2.24)	*0.61*
Disagree	1 (4.3%)	0 (0.0%)	5 (9.4%)	Ref.	
**Attitude about regular cannabis use by youth**					
Approve	11 (47.8%)	2 (28.6%)	11 (20.4%)	2.20 (0.84, 3.55)	*0.001*
Neither/nor	7 (30.4%)	4 (57.1%)	7 (13.0%)	2.30 (0.77, 3.82)	*0.003*
Disapprove	5 (21.7%)	1 (14.3%)	36 (66.7%)	Ref.	
**Perceived risk related to youth regular cannabis use**					
Mod/high risk	10 (43.5%)	4 (57.1%)	38 (70.4%)	-1.49 (-2.60, -0.37)	*0.009*
Do not know	0 (0.0%)	0 (0.0%)	5 (9.3%)	--[C]	--
No/low risk	13 (56.5%)	3 (42.9%)	11 (20.4%)	Ref.	
**Perceived health benefit related to youth regular cannabis use**					
Mod/high benefit	15 (65.2%)	0 (0.0%)	17 (31.5%)	1.05 (0.03, 2.07)	*0.04*

Do not know	1 (4.3%)	1 (14.3%)	8 (14.8%)	-0.57 (-2.36, 1.20)	*0.53*
No/low benefit	7 (30.4%)	6 (85.7%)	29 (53.7%)	Ref	
**State-level CL status**					
RCL	12 (52.2%)	2 (28.6%)	21 (38.9%)	0.17 (-0.25, 0.89)	*0.26*
MCL	8 (34.8%)	4 (57.1%)	21 (38.9%)	0.06 (-0.45, 0.66)	*0.70*
No-CL	3 (13.0%)	1 (14.3%)	12 (22.2%)	Ref.	

Note: With the goal of identifying variables for inclusion in our multivariable regression model, separate exploratory ordinal regression models were conducted for each variable shown in this table examining intention to use cannabisas the outcome variable and age, sex, caregiver education, and site as covariates/regressors of no-interest. Factors showing statistical significance at 2-sided p < 0.05 that were not collinear with each other were selected for inclusion in the main multilevel ordinal regression model presented in table 3. [A]Total psychiatric problem score: This is a diagnostic variable we created to index total psychiatric problems for each participant, calculated by coding each category as 1 = present and 0 = absent and summing across categories. We had planned to run regression analyses examining factors associated with intention to use CBD but there was insufficient variability in responses for an appropriately powered analysis, as only 3 youth respondents responded definitely or probably to this item. [B]Past year medical cannabisuse by a household member: This analysis was restricted to the subset of participants who were living in states with MCL or RCL + MCL, with participants in No-CL states excluded. [C]Among youth respondents with "definitely", "probably", and "possibly" intentions to use marijuana, 0.0% responded with "do not know" to question about perceived risk for harm from regular cannabisuse by youth. Abbreviations: CBD: cannabidiol; MM: medical marijuana; MJ: marijuana; No-CL: No cannabislaws; MCL: Medical CannabisLaws; RCL: Recreational CannabisLaw; Marijuana-related medical beliefs: Cannabisis a safe and effective treatment for pediatric mental health conditions; CBD-related medical beliefs: CBD is a safe and effective treatment for pediatric mental health conditions; MEEQ-B positive exp: MEEQ-B negative exp

eTable S5. Sensitivity Test: Leave-One-Out Validation (LOOV) Site Analysis Rerunning Multinominal and Linear Regression Models Examining Marijuana-related Attitudes, Perceptions, and Expectancies Using Respondent Group and State-Level Cannabis Law Status As Between-Subject Factors, Covarying for Sex and Parent Education, in US Youth Receiving Treatment for Mood Disorders and Their Parents, Using *n-1* Samples, Excluding Each Site (*n*).

A. Respondent Group Comparison

	**Group comparison:** Main Effect of Youth vs. Parents					
	LOOV Site Removed:					
	Site 1	Site 2	Site 3	Site 4	Site 5	Site 6
**Cannabis is a safe & effective txt**						
Agree	NS	NS	NS	NS	NS	NS
Neither/nor	NS	NS	NS	NS	NS	256.59***
Disagree	Ref.	Ref.	Ref.	Ref.	Ref.	Ref.

**Attitude about regular cannabis use by youth**						
Approve	--[X]	298.54***	--[X]	--[X]	295.35***	267.40***
Neither/nor	4.26*	5.04*	5.24*	4.85*	5.61*	NS
Disapprove	Ref.	Ref.	Ref.	Ref.	Ref.	Ref.
**Perceived risk for harm related to regular cannabis use by youth**						
Mod/high risk	4.77*	3.30±	3.95*	3.90*	6.67**	NS
Do not know	1.16	--[X]	1.37	1.18	NS	--[X]
No/low risk	Ref.	Ref.	Ref.	Ref.	Ref.	Ref.
**Perceived benefit for mental health related to regular cannabis use by youth**						
Mod/high benefit	6.26*	3.69±	5.58*	4.25*	5.53*	4.21*
Do not know	--[X]	--[X]	--[X]	--[X]	--[X]	--[X]
No/low benefit	Ref.	Ref.	Ref.	Ref.	Ref.	Ref.

B. State CL Status Comparison

	**State CL Status comparison**: Main Effect															
	LOOV Site Removed:															
	Site 1			Site 2			Site 3			Site 4			Site 5			
	MCL vs No-CL	No-CL vs RCL	MCL vs RCL	MCL vs No-CL	No-CL vs RCL	MCL vs RCL	MCL vs No-CL	No-CL vs RCL	MCL vs RCL	MCL vs No-CL	No-CL vs RCL	MCL vs RCL	MCL vs No-CL	No-CL vs RCL	MCL vs RCL	MCL vs No-CL
**Cannabis is a safe & effective txt**																
Agree	NS	NS	NS	NS	NS	NS	NS	--[X]	NS	182.06***	NS	255.67***	NS	NS	NS	NS
Neither/nor	110.87***	161.91***	NS	148.54***	164.26***	NS	--[X]	--[X]	2.65±	--[X]	--[X]	178.20***	149.92***	157.19***	NS	150.81***
Disagree	Ref.	Ref.	Ref.	Ref.	Ref.	Ref.	Ref.	Ref.	Ref.	Ref.	Ref.	Ref.	Ref.	Ref.	Ref.	Ref.
**Attitude about regular cannabis**																

**use by youth**																
Approve	NS	--[X]	NS	--[X]	--[X]	624.10***	135.19***	NS	--[X]	--[X]	NS	--[X]	--[X]	--[X]	700.68***	567.28***
Neither/nor	--[X]	NS	--[X]	NS	NS	NS	--[X]	NS	--[X]	NS	NS	NS	NS	NS	NS	NS
Disapprove	Ref.	Ref.	Ref.	Ref.	Ref.	Ref.	Ref.	Ref.	Ref.	Ref.	Ref.	Ref.	Ref.	Ref.	Ref.	Ref.
**Perceived risk for harm related to regular cannabis use by youth**																
Mod/high risk	NS	NS	NS	NS	NS	NS	--[X]	209.48***	NS	NS	NS	NS	NS	NS	NS	NS
Do not know	138.49***	NS	159.54***	172.71***	--[X]	NS	--[X]	--[X]	206.61***	--[X]	NS	--[X]	176.30***	NS	234.62***	176.14***
No/low risk	Ref.	Ref.	Ref.	Ref.	Ref.	Ref.	Ref.	Ref.	Ref.	Ref.	Ref.	Ref.	Ref.	Ref.	Ref.	Ref.
**Perceived benefit for mental health related to regular cannabis use by youth**																
Mod/high benefit	--[X]	NS	--[X]	NS	NS	NS	--[X]	--[X]	NS	NS	NS	NS	NS	NS	NS	NS
Do not know	--[X]	--[X]	NS	--[X]	--[X]	NS	--[X]	--[X]	NS	--[X]	--[X]	NS	--[X]	--[X]	NS	--[X]

No/low benefit	Ref.	Ref.	Ref.	Ref.	Ref.	Ref.	Ref.	Ref.	Ref.	Ref.	Ref.	Ref.	Ref.	Ref.	Ref.	Ref.

C. Group-By-State-CL-Status Comparison

	**Group-By-State-CL-Status Comparison:** Interaction Effect (Youth vs. Parents)																	
	LOOV Site Removed:																	
	Site 1			Site 2			Site 3			Site 4			Site 5			Site 6		
	MCL vs No-CL	No-CL vs RCL	MCL vs RCL	MCL vs No-CL	No-CL vs RCL	MCL vs RCL	MCL vs No-CL	No-CL vs RCL	MCL vs RCL	MCL vs No-CL	No-CL vs RCL	MCL vs RCL	MCL vs No-CL	No-CL vs RCL	MCL vs RCL	MCL vs No-CL	No-CL vs RCL	MCL vs RCL
**Cannabis is a safe & effective txt**																		
Agree	NS	NS	NS	NS	NS	NS	--[X]	--[X]	NS	--[X]	NS	--[X]	NS	NS	NS	NS	NS	NS
Neither/nor	--[X]	--[X]	NS	--[X]	--[X]	NS	--[X]	--[X]	NS	--[X]	--[X]	199.71***	--[X]	--[X]	NS	--[X]	--[X]	--[X]
Disagree	Ref.	Ref.	Ref.	Ref.	Ref.	Ref.	Ref.	Ref.	Ref.	Ref.	Ref.	Ref.	Ref.	Ref.	Ref.	Ref.	Ref.	Ref.
**Attitude about regular cannabis use by youth**																		
Approve	--[X]	--[X]	--[X]	--[X]	--[X]	--[X]	--[X]	--[X]	--[X]	--[X]	--[X]	--[X]	--[X]	--[X]	--[X]	--[X]	--[X]	--[X]
Neither/nor	--[X]	NS	--[X]	NS	NS	NS	--[X]	--[X]	NS	NS	NS	NS	NS	NS	NS	NS	NS	NS
Disapprove	Ref.	Ref.	Ref.	Ref.	Ref.	Ref.	Ref.	Ref.	Ref.	Ref.	Ref.	Ref.	Ref.	Ref.	Ref.	Ref.	Ref.	Ref.
**Perceived risk for harm related to regular cannabis use by youth**																		

Mod/high risk	NS	NS	NS	NS	NS	NS	--[X]	--[X]	NS	NS	NS	NS	NS	3.70±	2.81±	NS	NS	NS
Do not know	--[X]	NS	--[X]	--[X]	--[X]	--[X]	--[X]	--[X]	--[X]	--[X]	NS	--[X]	--[X]	NS	--[X]	--[X]	--[X]	--[X]
No/low risk	Ref.	Ref.	Ref.	Ref.	Ref.	Ref.	Ref.	Ref.	Ref.	Ref.	Ref.	Ref.	Ref.	Ref.	Ref.	Ref.	Ref.	Ref.
**Perceived benefit for mental health related to regular cannabis use by youth**																		
Mod/high benefit	NS	NS	--[X]	NS	NS	NS	--[X]	--[X]	NS	NS	NS	NS	NS	NS	NS	NS	NS	NS
Do not know	--[X]	--[X]	---[X]	--[X]	--[X]	--[X]	--[X]	--[X]	NS	--[X]	--[X]	--[X]	--[X]	--[X]	--[X]	--[X]	--[X]	--[X]
No/low benefit	Ref.	Ref.	Ref.	Ref.	Ref.	Ref.	Ref.	Ref.	Ref.	Ref.	Ref.	Ref.	Ref.	Ref.	Ref.	Ref.	Ref.	Ref.

Note: For this sensitivity test, Leave-One-Out-Validation (LOOV) analyses were performed rerunning the main analysis in subsamples of respondents that excluded all respondents from each site (n-1 samples). Each site listed is the site that was left out from the sample for each respective analysis. The analytic sample for each LOOV analysis was as follows: site 1 (n=123), site 2 (n=131), site 3 (n=119), site 4 (n=120), site 5 (n=138), and site 6 (n=125). Multinomial logistic regression models used medical beliefs, attitudes, risk perceptions, health benefit perceptions as outcome variables (reference group for each variable shown above) with respondent group membership and state-level ML status as between subject factor covarying for sex and parent education. Results are presented separately for Respondent Group Comparisons (**A**), State-level ML Status Comparisons (**B**), and Group-By-State-ML Status Comparisons (**C**). Results from the multinomial logistic regression models are presented as Wald Chi-Squared Test statistics. Statistical significance is shown with ± for $p < 0.10$, * for $p < 0.05$, ** for $p < 0.01$, and *** for $p < 0.001$. [X]Comparison had dependent variable levels in one of the comparator subpopulations with zero frequencies. Abbreviations: CBD: cannabidiol; MM: medical marijuana; MJ: marijuana; No-CL: No cannabislaws; MCL: Medical CannabisLaws; RCL: Recreational CannabisLaw; Marijuana-related medical beliefs: Cannabisis a safe and effective treatment for pediatric mental health conditions; CBD-related medical beliefs: CBD is a safe and effective treatment for pediatric mental health conditions

eTable S6. Sensitivity Test: Subgroup Analysis Rerunning Multinominal and Linear Regression Models With Respondent Group and State-Level CannabisLaw Status As Between-Subject Factors, Covarying for Sex and Parent Education, in a Subgroup Sample of US Youth Receiving Treatment for Mood Disorders Who Meet Clinical Threshold/Criteria for Current Depression and Parents

	**Group comparison:** Main Effect	**State CL Status comparison**: Main Effect			**Group-By-State-CL-Status Comparison:** Interaction Effect		
					Youth vs. Parents		
	Youth vs. Parents	MCL vs. No-CL	No-CL vs. RCL	MCL vs. RCL	MCL vs. No-CL	No-CL vs. RCL	MCL vs. RCL
	Wald Chi-Squared Test Statistic	Wald Chi-Squared Test Statistic	Wald Chi-Squared Test Statistic	Wald Chi-Squared Test Statistic	Wald Chi-Squared Test Statistic	Wald Chi-Squared Test Statistic	Wald Chi-Squared Test Statistic
**MC is a safe & effective txt**							
Agree	0.80	2.39	2.82±	0.09	--X	--X	0.22
Neither/nor	1.49	--X	--X	2.93±	--X	--X	2.11
Disagree	Ref.	Ref.	Ref.	Ref.	Ref.	Ref.	Ref.
**CBD is a safe & effective txt**							
Agree	0.01	0.19	1.43	0.35	--X	--X	0.02
Neither/nor	0.01	2.45±	0.72	0.64	--X	--X	0.01
Disagree	Ref.	Ref.	Ref.	Ref.	Ref.	Ref.	Ref.
**Attitude about regular cannabis use by youth**							
Approve	--X	0.02	--X	--X	--X	--X	--X
Neither/nor	4.76*	0.05	0.17	0.06	--X	--X	0.05
Disapprove	Ref.	Ref.	Ref.	Ref.	Ref.	Ref.	Ref.
**Attitude about MC use by youth**							
Approve	--X	0.08	1.95	2.65	--X	--X	--X
Neither/nor	--X	0.01	0.19	0.24	83.88***	0.26	--X
Disapprove	Ref.	Ref.	Ref.	Ref.	Ref.	Ref.	Ref.
**Attitude about CBD use by youth**							
Approve	237.98***	1.42	1.53	5.38*	--X	--X	--X

Neither/nor	143.71***	0.08	1.16	1.91	97.29***	0.83	152.66***
Disapprove	Ref.	Ref.	Ref.	Ref.	Ref.	Ref.	Ref.
**Perceived risk for harm related to regular cannabis use by youth**							
Mod/high risk	1.11	0.15	0.42	0.06	0.32	0.10	0.09
Do not know	--[X]	0.01	0.39	--[X]	--[X]	--[X]	--[X]
No/low risk	Ref.	Ref.	Ref.	Ref.	Ref.	Ref.	Ref.
**Perceived risk for harm related to MC use by youth**							
Mod/high risk	3.69±	0.07	0.09	0.00	0.00	0.36	0.58
Do not know	--[X]	0.46	--[X]	--[X]	--[X]	--[X]	--[X]
No/low risk	Ref.	Ref.	Ref.	Ref.	Ref.	Ref.	Ref.
**Perceived risk for harm related to CBD use by youth**							
Mod/high risk	0.10	0.17	0.49	1.33	--[X]	--[X]	0.48
Do not know	--[X]	0.87	--[X]	--[X]	--[X]	--[X]	--[X]
No/low risk	Ref.	Ref.	Ref.	Ref.	Ref.	Ref.	Ref.
**Perceived benefit for mental health related to regular cannabis use by youth**							
Mod/high benefit	3.77±	0.93	0.00	0.96	0.12	0.25	0.01
Do not know	--[X]	--[X]	--[X]	--[X]	--[X]	--[X]	--[X]
No/low benefit	Ref.	Ref.	Ref.	Ref.	Ref.	Ref.	Ref.
**Perceived benefit for mental health related to MC use by youth**							
Mod/high benefit	4.39*	0.90	0.08	1.57	0.00	0.01	0.01
Do not know	--[X]	--[X]	--[X]	--[X]	--[X]	--[X]	--[X]
No/low benefit	Ref.	Ref.	Ref.	Ref.	Ref.	Ref.	Ref.
**Perceived benefit for mental health related to CBD use by youth**							

Mod/high benefit	1.54	0.01	2.28	2.45	0.31	0.55	0.05
Do not know	--[X]	--[X]	--[X]	--[X]	--[X]	--[X]	--[X]
No/low benefit	Ref.	Ref.	Ref.	Ref.	Ref.	Ref.	Ref.
**Expectancies**	**Group Comparison**: Youth vs. Parent	**State CL Status Comparison**: No-CL (1) vs. MCL (2) vs. RCL (3)					
	Beta (95%CI)	Beta (95%CI)					
MEEQ-B positive exp.	-0.15 (-0.47, 0.17)	0.07 (-0.11, 0.26)					
MEEQ-B negative exp.	0.24 (-0.11, 0.59)	0.05 (-0.15, 0.25)					

Note: For this sensitivity test, subgroup analyses were run in a sample that included all Parent Respondents and a Subset of Youth Respondents Who met clinical threshold/criteria for Current Depressive Disorder based upon PHQ-2 score $\geq$ 3. We did the following: Multinomial logistic regression models used medical beliefs, attitudes, risk perceptions, health benefit perceptions as outcome variables (reference group for each variable shown above) with respondent group membership and state-level ML status as between subject factor covarying for sex and parent education. Linear regression models used MEEQ-B positive and negative expectancy scores as outcome variables with respondent group membership and state-level ML status as between-subjects factor covarying for sex and caregiver education. Results from the multinomial logistic regression models are presented as Wald Chi-Squared Test statistics. Results from the linear regression models are presented as t-test statistics. Statistical significance is shown with ± for $p < 0.10$, * for $p < 0.05$, ** for $p < 0.01$, and *** for $p < 0.001$. [X]Comparison had dependent variable levels in one of the comparator subpopulations with zero frequencies. Abbreviations: CBD: cannabidiol; MM: medical marijuana; MJ: marijuana; No-CL: No cannabislaws; MCL: Medical CannabisLaws; RCL: Recreational CannabisLaw; Marijuana-related medical beliefs: Cannabisis a safe and effective treatment for pediatric mental health conditions; CBD-related medical beliefs: CBD is a safe and effective treatment for pediatric mental health conditions; MEEQ-B positive exp: MEEQ-B; negative exp:

eTable S7. Sensitivity Test: Subgroup Analysis Rerunning Multinominal and Linear Regression Models With Respondent Group and State-Level CannabisLaw Status As Between-Subject Factors, Covarying for Sex and Parent Education, in US Youth Receiving Treatment for Mood Disorders Who Meet Clinical Threshold/Criteria for Current Anxiety Disorder and Parents

	**Group comparison:** Main Effect	**State CL Status comparison**: Main Effect			**Group-By-State-CL-Status Comparison:** Interaction Effect		
					Youth vs. Parents		
	Youth vs. Parents	MCL vs. No-CL	No-CL vs. RCL	MCL vs. RCL	MCL vs. No-CL	No-CL vs. RCL	MCL vs. RCL

	Wald Chi-Squared Test Statistic	Wald Chi-Squared Test Statistic	Wald Chi-Squared Test Statistic	Wald Chi-Squared Test Statistic	Wald Chi-Squared Test Statistic	Wald Chi-Squared Test Statistic	Wald Chi-Squared Test Statistic
**MC is a safe & effective txt**							
Agree	1.20	2.43±	3.81±	0.01	--[X]	--[X]	--[X]
Neither/nor	1.95	--[X]	--[X]	3.13±	--[X]	--[X]	--[X]
Disagree	Ref.	Ref.	Ref.	Ref.	Ref.	Ref.	Ref.
**CBD is a safe & effective txt**							
Agree	0.08	0.13	2.01	0.69	--[X]	--[X]	--[X]
Neither/nor	0.00	2.50±	0.75	0.66	--[X]	--[X]	149.28***
Disagree	Ref.	Ref.	Ref.	Ref.	Ref.	Ref.	Ref.
**Attitude about regular cannabis use by youth**							
Approve	0.03	0.02	0.00	0.02	0.02	0.00	0.03
Neither/nor	6.33**	0.02	0.16	0.05	--[X]	--[X]	0.16
Disapprove	Ref.	Ref.	Ref.	Ref.	Ref.	Ref.	Ref.
**Attitude about MC use by youth**							
Approve	--[X]	0.04	2.21	2.64	--[X]	--[X]	--[X]
Neither/nor	613.09***	0.00	0.58	0.51	0.20	0.32	0.01
Disapprove	Ref.	Ref.	Ref.	Ref.	Ref.	Ref.	Ref.
**Attitude about CBD use by youth**							
Approve	--[X]	1.35	1.77	5.62*	--[X]	--[X]	--[X]
Neither/nor	627.49***	0.05	1.57	2.25	--[X]	0.80	0.09
Disapprove	Ref.	Ref.	Ref.	Ref.	Ref.	Ref.	Ref.
**Perceived risk for harm related to regular cannabis use by youth**							
Mod/high risk	1.34	0.14	0.87	0.91	0.06	1.30	1.24
Do not know	--[X]	--[X]	0.05	--[X]	--[X]	--[X]	--[X]
No/low risk	Ref.	Ref.	Ref.	Ref.	Ref.	Ref.	Ref.

**Perceived risk for harm related to MC use by youth**							
Mod/high risk	4.85*	0.06	0.08	0.00	0.24	0.01	0.52
Do not know	--[X]	0.57	--[X]	--[X]	--[X]	--[X]	--[X]
No/low risk	Ref.	Ref.	Ref.	Ref.	Ref.	Ref.	Ref.
**Perceived risk for harm related to CBD use by youth**							
Mod/high risk	0.54	0.18	0.39	1.17	--[X]	--[X]	0.07
Do not know	--[X]	1.02	--[X]	--[X]	--[X]	--[X]	--[X]
No/low risk	Ref.	Ref.	Ref.	Ref.	Ref.	Ref.	Ref.
**Perceived benefit for mental health related to regular cannabis use by youth**							
Mod/high benefit	2.38	0.79	0.05	1.13	0.69	0.04	0.54
Do not know	--[X]	--[X]	--[X]	--[X]	--[X]	--[X]	--[X]
No/low benefit	Ref.	Ref.	Ref.	Ref.	Ref.	Ref.	Ref.
**Perceived benefit for mental health related to MC use by youth**							
Mod/high benefit	5.15*	0.65	0.25	1.68	0.01	0.00	0.03
Do not know	--[X]	--[X]	--[X]	--[X]	--[X]	--[X]	--[X]
No/low benefit	Ref.	Ref.	Ref.	Ref.	Ref.	Ref.	Ref.
**Perceived benefit for mental health related to CBD use by youth**							
Mod/high benefit	0.20	0.00	2.67	2.56	0.06	1.63	1.33
Do not know	--[X]	--[X]	--[X]	--[X]	--[X]	--[X]	--[X]
No/low benefit	Ref.	Ref.	Ref.	Ref.	Ref.	Ref.	Ref.
**Expectancies**	**Group Comparison**: Youth vs. Parent	**State CL Status Comparison**: No-CL (1) vs. MCL (2) vs. RCL (3)					
	Beta (95%CI)	Beta (95%CI)					
MEEQ-B positive exp.	-0.12 (-0.40, 0.16)	0.00 (-0.17, 0.17)					

MEEQ-B negative exp.	0.20 (-0.10, 0.50)	0.02 (-0.16, 0.19)			

Note: For this sensitivity test, subgroup analyses were run in a sample that included all Parent Respondents and a Subset of Youth Respondents Who met clinical threshold/criteria for Current Anxiety Disorder based upon GAD-2 score $\geq$ 3 (n=105). We did the following: Multinomial logistic regression models used medical beliefs, attitudes, risk perceptions, health benefit perceptions as outcome variables (reference group for each variable shown above) with respondent group membership and state-level ML status as between subject factor covarying for sex and parent education. Linear regression models used MEEQ-B positive and negative expectancy scores as outcome variables with respondent group membership and state-level ML status as between-subjects factor covarying for sex and caregiver education. Results from the multinomial logistic regression models are presented as Wald Chi-Squared Test statistics. Results from the linear regression models are presented as t-test statistics. Statistical significance is shown with ± for $p < 0.10$, * for $p < 0.05$, ** for $p < 0.01$, and *** for $p < 0.001$. [X]Comparison had dependent variable levels in one of the comparator subpopulations with zero frequencies.
Abbreviations: CBD: cannabidiol; MM: medical marijuana; MJ: marijuana; No-CL: No cannabislaws; MCL: Medical CannabisLaws; RCL: Recreational CannabisLaw; Marijuana-related medical beliefs: Cannabisis a safe and effective treatment for pediatric mental health conditions; CBD-related medical beliefs: CBD is a safe and effective treatment for pediatric mental health conditions; MEEQ-B positive exp: MEEQ-B; negative exp:

eTable S8. Sensitivity Test: Subgroup Analysis Rerunning Multinominal and Linear Regression Models With Respondent Group and State-Level Cannabis Law Status As Between-Subject Factors, Covarying for Sex and Parent Education, in US Youth Receiving Treatment for Mood Disorders Who Report No Lifetime History of Alcohol or Drug Use Problems and Parents

	**Group comparison:** Main Effect	**State CL Status comparison**: Main Effect			**Group-By-State-CL-Status Comparison:** Interaction Effect		
					Youth vs. Parents		
	Youth vs. Parents	MCL vs. No-CL	No-CL vs. RCL	MCL vs. RCL	MCL vs. No-CL	No-CL vs. RCL	MCL vs. RCL
	Wald Chi-Squared Test Statistic	Wald Chi-Squared Test Statistic	Wald Chi-Squared Test Statistic	Wald Chi-Squared Test Statistic	Wald Chi-Squared Test Statistic	Wald Chi-Squared Test Statistic	Wald Chi-Squared Test Statistic
**MC is a safe & effective txt**							
Agree	0.42	1.68	1.29	0.19	0.41	0.93	0.08
Neither/nor	2.42	143.46***	145.98***	3.09±	--[X]	--[X]	2.25
Disagree	Ref.	Ref.	Ref.	Ref.	Ref.	Ref.	Ref.
**CBD is a safe & effective txt**							
Agree	0.09	0.39	1.20	0.14	0.02	0.77	1.03

Neither/nor	0.02	141.63***	176.00***	0.67	--X	--X	0.53
Disagree	Ref.	Ref.	Ref.	Ref.	Ref.	Ref.	Ref.
**Attitude about regular cannabis use by youth**							
Approve	--X	--X	0.04	--X	--X	--X	--X
Neither/nor	3.87*	0.07	0.01	0.04	0.49	0.29	0.07
Disapprove	Ref.	Ref.	Ref.	Ref.	Ref.	Ref.	Ref.
**Attitude about MC use by youth**							
Approve	4.48*	0.01	2.34	2.67	0.21	0.47	1.91
Neither/nor	3.00±	0.25	0.57	0.34	0.00	0.00	0.00
Disapprove	Ref.	Ref.	Ref.	Ref.	Ref.	Ref.	Ref.
**Attitude about CBD use by youth**							
Approve	1.73	0.04	3.28±	5.05*	2.11	0.00	2.34
Neither/nor	0.32	0.10	2.09	1.80	0.10	0.28	0.79
Disapprove	Ref.	Ref.	Ref.	Ref.	Ref.	Ref.	Ref.
**Perceived risk for harm related to regular cannabis use by youth**							
Mod/high risk	4.02*	1.04	1.90	0.12	0.73	2.56	0.68
Do not know	0.93	0.34	--X	203.86***	--X	--X	--X
No/low risk	Ref.	Ref.	Ref.	Ref.	Ref.	Ref.	Ref.
**Perceived risk for harm related to MC use by youth**							
Mod/high risk	4.53*	0.38	0.27	0.03	3.40±	2.81±	0.07
Do not know	317.92***	1.09	125.10***	400.63***	2.53	98.06***	--X
No/low risk	Ref.	Ref.	Ref.	Ref.	Ref.	Ref.	Ref.
**Perceived risk for harm related to CBD use by youth**							
Mod/high risk	0.46	0.02	1.24	1.23	0.86	0.13	0.41
Do not know	333.26***	0.67	114.84***	274.38***	1.29	107.75***	--X

No/low risk	Ref.	Ref.	Ref.	Ref.	Ref.	Ref.	Ref.
**Perceived benefit for mental health related to regular cannabis use by youth**							
Mod/high benefit	4.66*	1.94	0.54	0.75	0.92	0.23	0.37
Do not know	--[X]	--[X]	--[X]	0.07	--[X]	--[X]	--[X]
No/low benefit	Ref.	Ref.	Ref.	Ref.	Ref.	Ref.	Ref.
**Perceived benefit for mental health related to MC use by youth**							
Mod/high benefit	9.20**	1.82	0.14	1.41	0.52	1.36	0.22
Do not know	--[X]	0.12	--[X]	0.16	--[X]	--[X]	--[X]
No/low benefit	Ref.	Ref.	Ref.	Ref.	Ref.	Ref.	Ref.
**Perceived benefit for mental health related to CBD use by youth**							
Mod/high benefit	0.56	0.33	0.49	2.09	0.23	0.01	0.51
Do not know	255.67***	169.17***	--[X]	391.98***	--[X]	--[X]	--[X]
No/low benefit	Ref.	Ref.	Ref.	Ref.	Ref.	Ref.	Ref.
**Expectancies**	**Group Comparison**: Youth vs. Parent	**State CL Status Comparison**: No-CL (1) vs. MCL (2) vs. RCL (3)					
	Beta (95%CI)	Beta (95%CI)					
MEEQ-B positive exp.	-0.05 (-0.33, 0.23)	0.07 (-0.11, 0.25)					
MEEQ-B negative exp.	0.10 (-0.18, 0.37)	0.12 (-0.06, 0.29)					

<u>Note</u>: For this sensitivity test, subgroup Analyses were run in a sample that included a subset of Youth Respondents Who Reported No Lifetime History of Alcohol or Drug Use Problems and Parents/Caregiver respondents who reported no lifetime history of alcohol or drug use problems in their youth offspring (n=132 respondents [75 youth and 57 parents]). We did the following: Multinomial logistic regression models used medical beliefs, attitudes, risk perceptions, health benefit perceptions as outcome variables (reference group for each variable shown above) with respondent group membership and state-level ML status as between subject factor covarying for sex and parent education. Linear regression models used MEEQ-B positive and negative expectancy scores as outcome variables with respondent group membership and state-level ML status as between-subjects factor covarying for sex and parent education. Results from the multinomial logistic regression models are presented as Wald Chi-Squared Test statistics. Results from the linear regression models are presented as t-test statistics. Statistical significance is shown with ± for $p < 0.10$, * for $p < 0.05$, ** for $p < 0.01$, and *** for $p < 0.001$. [X]Comparison had dependent variable levels in one of the comparator subpopulations with zero frequencies. <u>Abbreviations</u>: CBD: cannabidiol; MM: medical marijuana; MJ: marijuana; No-CL: No

cannabislaws; MCL: Medical CannabisLaws; RCL: Recreational CannabisLaw; Marijuana-related medical beliefs: Cannabisis a safe and effective treatment for pediatric mental health conditions; CBD-related medical beliefs: CBD is a safe and effective treatment for pediatric mental health conditions; MEEQ-B positive exp: MEEQ-B; negative exp:

eTable S9. Sensitivity Test: Subgroup Analysis Rerunning Multinominal and Linear Regression Models With Respondent Group and State-Level CannabisLaw Status As Between-Subject Factors, Covarying for Sex and Parent Education, in Parent-Child Dyads of US Youth Receiving Treatment for Mood Disorders and Parents

	**Group comparison:** Main Effect	**State CL Status comparison**: Main Effect			**Group-By-State-CL-Status Comparison:** Interaction Effect		
					Youth vs. Parents		
	Youth vs. Parents	MCL vs. No-CL	No-CL vs. RCL	MCL vs. RCL	MCL vs. No-CL	No-CL vs. RCL	MCL vs. RCL
	Wald Chi-Squared Test Statistic	Wald Chi-Squared Test Statistic	Wald Chi-Squared Test Statistic	Wald Chi-Squared Test Statistic	Wald Chi-Squared Test Statistic	Wald Chi-Squared Test Statistic	Wald Chi-Squared Test Statistic
**MC is a safe & effective txt**							
Agree	0.00	--X	--X	0.90	--X	--X	0.00
Neither/nor	0.01	--X	--X	0.01	--X	--X	0.02
Disagree	Ref.	Ref.	Ref.	Ref.	Ref.	Ref.	Ref.
**CBD is a safe & effective txt**							
Agree	--X	--X	--X	--X	--X	--X	--X
Neither/nor	--X	0.67	--X	--X	--X	--X	--X
Disagree	Ref.	Ref.	Ref.	Ref.	Ref.	Ref.	Ref.
**Attitude about regular cannabis use by youth**							

Approve	--X	--X	--X	--X	--X	--X	--X
Neither/nor	--X	--X	--X	--X	--X	--X	--X
Disapprove	Ref.	Ref.	Ref.	Ref.	Ref.	Ref.	Ref.
**Attitude about MC use by youth**							
Approve	--X	--X	--X	3.56±	--X	--X	--X
Neither/nor	--X	--X	--X	0.52	--X	--X	--X
Disapprove	Ref.	Ref.	Ref.	Ref.	Ref.	Ref.	Ref.
**Attitude about CBD use by youth**							
Approve	--X	--X	--X	5.11*	--X	--X	--X
Neither/nor	--X	--X	--X	2.17	--X	--X	--X
Disapprove	Ref.	Ref.	Ref.	Ref.	Ref.	Ref.	Ref.
**Perceived risk for harm related to regular cannabis use by youth**							
Mod/high risk	0.17	--X	--X	0.07	--X	--X	0.00
Do not know	--X	--X	--X	--X	--X	--X	--X
No/low risk	Ref.	Ref.	Ref.	Ref.	Ref.	Ref.	Ref.
**Perceived risk for harm related to MC use by youth**							
Mod/high risk	2.03	--X	--X	0.20	--X	--X	0.34
Do not know	--X	--X	--X	--X	--X	--X	--X
No/low risk	Ref.	Ref.	Ref.	Ref.	Ref.	Ref.	Ref.
**Perceived risk for harm related to CBD use by youth**							
Mod/high risk	0.48	--X	--X	0.13	--X	--X	0.03
Do not know	--X	--X	--X	--X	--X	--X	--X
No/low risk	Ref.	Ref.	Ref.	Ref.	Ref.	Ref.	Ref.
**Perceived benefit for mental health related to regular cannabis use by youth**							

Mod/high benefit	2.99±	--X	--X	0.16	--X	--X	0.02
Do not know	--X	--X	--X	--X	--X	--X	--X
No/low benefit	Ref.	Ref.	Ref.	Ref.	Ref.	Ref.	Ref.
**Perceived benefit for mental health related to MC use by youth**							
Mod/high benefit	1.54	--X	--X	3.24±	--X	--X	0.15
Do not know	--X	--X	--X	--X	--X	--X	--X
No/low benefit	Ref.	Ref.	Ref.	Ref.	Ref.	Ref.	Ref.
**Perceived benefit for mental health related to CBD use by youth**							
Mod/high benefit	0.26	--X	--X	2.70±	--X	--X	1.10
Do not know	--X	--X	--X	--X	--X	--X	--X
No/low benefit	Ref.	Ref.	Ref.	Ref.	Ref.	Ref.	Ref.
**Expectancies**	**Group Comparison**: Youth vs. Parent	**State CL Status Comparison**: No-CL (1) vs. MCL (2) vs. RCL (3)					
	Beta (95%CI)	Beta (95%CI)					
MEEQ-B positive exp.	0.06 (-0.29, 0.41)	0.37 (0.06, 0.68)*					
MEEQ-B negative exp.	-0.13 (-0.57, 0.32)	-0.07 (-0.46, 0.32)					

Note: For this sensitivity test, subgroup Analyses were run in a sample that included a subset of US Youth Respondents and Matched Parents/Caregiver respondents who were Parent-Child Dyads (n=30 dyads [30 youth and 30 parents]). We did the following: Multinomial logistic regression models used medical beliefs, attitudes, risk perceptions, health benefit perceptions as outcome variables (reference group for each variable shown above) with respondent group membership and state-level ML status as between subject factor covarying for sex and parent education. Linear regression models used MEEQ-B positive and negative expectancy scores as outcome variables with respondent group membership and state-level ML status as between-subjects factor covarying for sex and parent education. Results from the multinomial logistic regression models are presented as Wald Chi-Squared Test statistics. Results from the linear regression models are presented as t-test statistics. Statistical significance is shown with ± for $p < 0.10$, * for $p < 0.05$, ** for $p < 0.01$, and *** for $p < 0.001$. XComparison had dependent variable levels in one of the comparator subpopulations with zero frequencies. Abbreviations: CBD: cannabidiol; MM: medical marijuana; MJ: marijuana; No-CL: No cannabis laws; MCL: Medical Cannabis Laws; RCL: Recreational Cannabis Law; Marijuana-related medical beliefs: Cannabis is a safe and effective treatment for pediatric mental health conditions; CBD-related medical beliefs: CBD is a safe and effective treatment for pediatric mental health conditions; MEEQ-B positive exp: MEEQ-B; negative exp:

Supplemental eFigure S1. Perceptions of moderate-to-great and uncertain/unknown mental health benefits and harms related to adolescent cannabis use across frequencies, methods of administration, and chemotypes and related to adolescent CBD use, as a function of respondent group

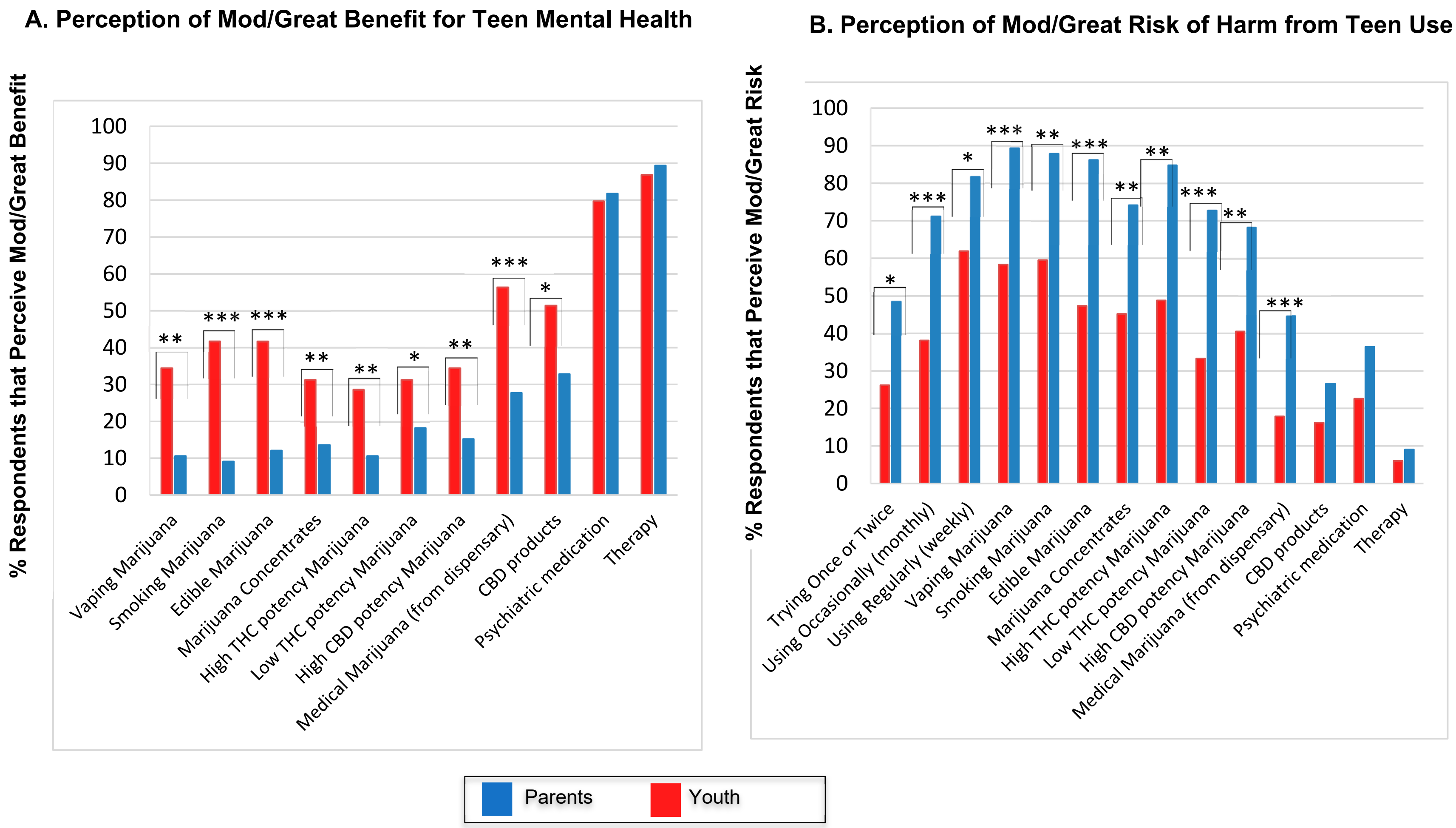

C. Perception of Unknown Benefit for Teen Mental Health

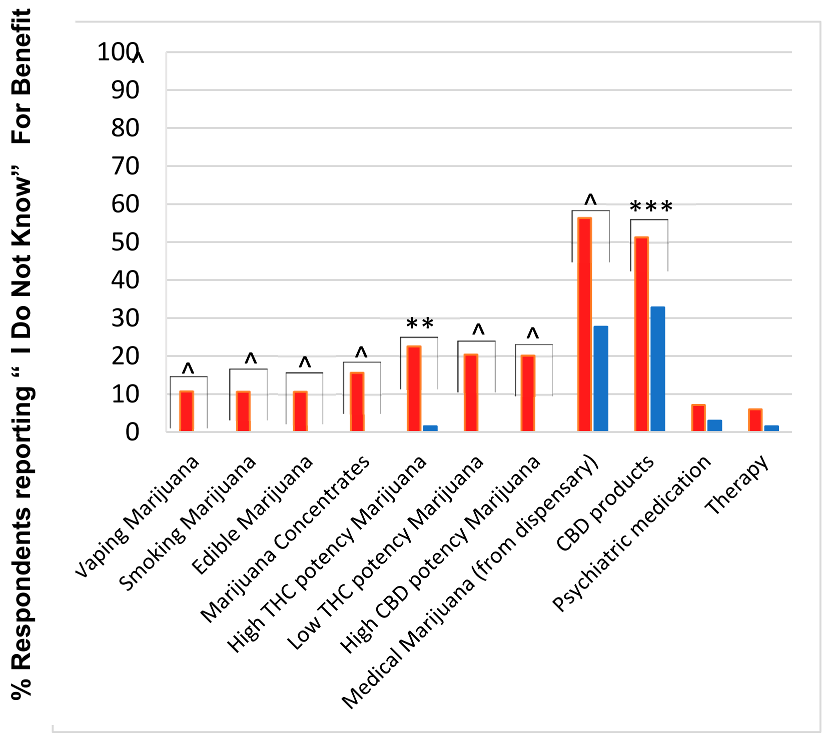

D. Perception of Unknown Risk of Harm from Teen Use

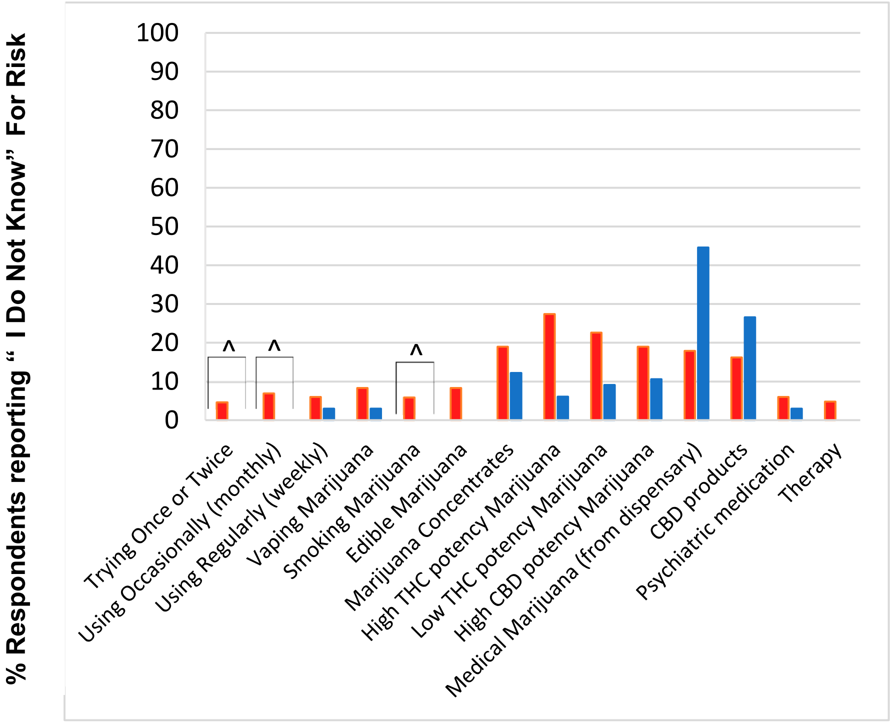

eFig S1. Caption: eFig S1 depicts group comparison of perceived mental health benefits and risks of harm related to adolescent cannabis use (across different frequencies, methods of administration, and chemotypes) and related to adolescent CBD product use, between youth with mood disorders and parent respondents. (**A**) Differences in the proportion of respondents that perceive *moderate-to-great benefit* for teen mental health related to product use; (**B**) Differences in the proportion of respondents that perceive *moderate-to-great risk* of harm related to teen product use; (**C**) Differences in the proportion of respondents who are *uncertain about benefits* and report "I don't know" when asked their perceptions about the benefits for mental health related to teen use of products; and (**D**) Differences in the proportion of respondents who are *uncertain about risks* and report "I don't know" when asked their perceptions about the risks for harm related to teen use of products. For each comparison, health benefit and risk perceptions for the use of psychiatric medications and engaging in psychotherapy are presented as comparators. All star'd (*) items are statistically significant differences between youth and parent respondent groups based upon multinomial logistic regression analyses using respondent group as a between-subject factor and covarying for sex, site, parent education. ***= $p<0.001$; **= $p<0.01$; *= $p<0.05$. ^ = Comparisons that had dependent variable levels in one of the comparator subpopulations with zero frequencies making covariate-adjusted comparisons incalculable. Youth respondents reported higher perceptions of moderate-to-great benefits to mental health and lower perceptions of moderate-to-great risks of harm related to adolescent cannabis use across different frequencies, methods of use, and chemotypes compared to parent respondents. Youth respondents also reported higher perceptions of moderate-to-great health benefits related to adolescent CBD product use compared to parent respondents. Regarding perceived uncertainty/unknown risk/benefit, the cannabinoid products that both youth and parent respondents were most likely to report "I don't know" when asked about perceived benefits and harms related to teen use were medical cannabis(obtained from a dispensary) and CBD products, respectively. In general, youth respondents

were more likely to report uncertainty (i.e., responding “I don’t know”) related to perceived health benefits from adolescent use of CBD products and cannabis across methods of administration and chemotypes compared to parent respondents. A subgroup of both youth and parent respondents reported uncertainty (i.e., responding “I don’t know”) related to perceived risks for harm from adolescent use of cannabis and CBD products, but no respondent group differences reached statistical significance. Parents exhibited higher uncertainty regarding risk perceptions related to medical cannabis treatment and CBD product use in general, although no statistical differences were seen between respondent groups.

eTable S10: STROBE Statement—checklist of items included in the current report of a cross-sectional study

	Item No.	Recommendation	Page No.
**Title and abstract**	1	(*a*) Indicate the study's design with a commonly used term in the title or the abstract	Page 1
		(*b*) Provide in the abstract an informative and balanced summary of what was done and what was found	Page 1
**Introduction**			
Background/rationale	2	Explain the scientific background and rationale for the investigation being reported	Pages 2-3
Objectives	3	State specific objectives, including any prespecified hypotheses	Page 3
**Methods**			
Study design	4	Present key elements of study design early in the paper	Pages 3-4
Setting	5	Describe the setting, locations, and relevant dates, including periods of recruitment, exposure, follow-up, and data collection	Pages 4-5
Participants	6	*Cross-sectional study*—Give the eligibility criteria, and the sources and methods of selection of participants	Page 4
		(*b*) *Cohort study*—For matched studies, give matching criteria and number of exposed and unexposed<br>*Case-control study*—For matched studies, give matching criteria and the number of controls per case	N/A
Variables	7	Clearly define all outcomes, exposures, predictors, potential confounders, and effect modifiers. Give diagnostic criteria, if applicable	Pages 4-6
Data sources/ measurement	8*	For each variable of interest, give sources of data and details of methods of assessment (measurement). Describe comparability of assessment methods if there is more than one group	Pages 4-5
Bias	9	Describe any efforts to address potential sources of bias	Page 6
Study size	10	Explain how the study size was arrived at	Page 4

Continued on next page

Quantitative variables	11	Explain how quantitative variables were handled in the analyses. If applicable, describe which groupings were chosen and why	Page 6
Statistical methods	12	(*a*) Describe all statistical methods, including those used to control for confounding	Page 6
		(*b*) Describe any methods used to examine subgroups and interactions	Page 6
		(*c*) Explain how missing data were addressed	Page 6
		(*d*) *Cohort study*—If applicable, explain how loss to follow-up was addressed<br>*Case-control study*—If applicable, explain how matching of cases and controls was addressed<br>*Cross-sectional study*—If applicable, describe analytical methods taking account of sampling strategy	Page 6
		(*e*) Describe any sensitivity analyses	Page 6
**Results**			
Participants	13*	(a) Report numbers of individuals at each stage of study—eg numbers potentially eligible, examined for eligibility, confirmed eligible, included in the study, completing follow-up, and analysed	Page 6
		(b) Give reasons for non-participation at each stage	Page 6
		(c) Consider use of a flow diagram	Page 6
Descriptive data	14*	(a) Give characteristics of study participants (eg demographic, clinical, social) and information on exposures and potential confounders	Pages 6-8, Table 1
		(b) Indicate number of participants with missing data for each variable of interest	Page 6
		(c) *Cohort study*—Summarise follow-up time (eg, average and total amount)	NA
Outcome data	15*	*Cohort study*—Report numbers of outcome events or summary measures over time	*N/A*
		Case-control study—Report numbers in each exposure category, or summary measures of exposure	*N/A*
		Cross-sectional study—Report numbers of outcome events or summary measures	Page 6
Main results	16	(*a*) Give unadjusted estimates and, if applicable, confounder-adjusted estimates and their precision (eg, 95% confidence interval). Make clear which confounders were adjusted for and why they were included	Pages 7-14, Tables 2-4, Supplemental eTables S3-4
		(*b*) Report category boundaries when continuous variables were categorized	Pages 7-14, Tables 2-4, Supplemental eTables S3-4
		(*c*) If relevant, consider translating estimates of relative risk into absolute risk for a meaningful time period	Pages 7-14, Tables 2-4, Supplemental eTables S3-4

Continued on next page

Other analyses	17	Report other analyses done—eg analyses of subgroups and interactions, and sensitivity analyses	Page 14, Supplemental eTables S5-9, eResults S3
**Discussion**			
Key results	18	Summarise key results with reference to study objectives	Pages 14-17
Limitations	19	Discuss limitations of the study, taking into account sources of potential bias or imprecision. Discuss both direction and magnitude of any potential bias	Pages 17-18
Interpretation	20	Give a cautious overall interpretation of results considering objectives, limitations, multiplicity of analyses, results from similar studies, and other relevant evidence	Pages 14-18
Generalisability	21	Discuss the generalisability (external validity) of the study results	Pages 14-18
**Other information**			
Funding	22	Give the source of funding and the role of the funders for the present study and, if applicable, for the original study on which the present article is based	Page 19

Notes on the STROBE checklist and guidelines: An Explanation and Elaboration article discusses each checklist item and gives methodological background and published examples of transparent reporting. The STROBE checklist is best used in conjunction with this article (freely available on the Web sites of PLoS Medicine at http://www.plosmedicine.org/, Annals of Internal Medicine at http://www.annals.org/, and Epidemiology at http://www.epidem.com/). Information on the STROBE Initiative is available at www.strobe-statement.org.

REFERENCES

1. Hammond CJ, Chaney A, Hendrickson B, Sharma P. Cannabis use among U.S. adolescents in the era of cannabislegalization: a review of changing use patterns, comorbidity, and health correlates. *International review of psychiatry (Abingdon, England).* 2020;32(3):221-234.
2. Gobbi G, Atkin T, Zytynski T, et al. Association of Cannabis Use in Adolescence and Risk of Depression, Anxiety, and Suicidality in Young Adulthood: A Systematic Review and Meta-analysis. *JAMA Psychiatry.* 2019;76(4):426-434.
3. Di Forti M, Sallis H, Allegri F, et al. Daily use, especially of high-potency cannabis, drives the earlier onset of psychosis in cannabis users. *Schizophr Bull.* 2014;40(6):1509-1517.
4. Torrealday O, Stein LA, Barnett N, et al. Validation of the CannabisEffect Expectancy Questionnaire-Brief. *J Child Adolesc Subst Abuse.* 2008;17(4):1-17.
5. Kroenke K, Spitzer RL, Williams JBW. The Patient Health Questionnaire-2: Validity of a Two-Item Depression Screener. *Medical Care.* 2003;41(11).
6. Plummer F, Manea L, Trepel D, McMillan D. Screening for anxiety disorders with the GAD-7 and GAD-2: a systematic review and diagnostic metaanalysis. *General hospital psychiatry.* 2016;39:24-31.
